# Supplementary material for: Noise promotes independent control of gamma oscillations and grid firing within recurrent attractor networks
Source: eLife. 2015 Jul 6;4:e06444. doi: 10.7554/eLife.06444 (PMC4508578; doi:10.7554/eLife.06444)
Supplement: Supplementary file 1. — Examples of spatial firing fields. (A-L) Top: Gridness score in the parameter space of the E and I synaptic strength scaling parameters (gE and gI respectively). Bottom: Firing fields of a single cell obtained by simulating animal movement, in the parameter region highlighted by black rectangle in the parameter space plot. Above each firing field is the estimated gridness score (left) and maximal firing rate in the firing field (right). Blank (white) locations in the parameter space are simulations that did not finish in the pre-specified time limit (5 hr). Noise level used in each set of simulations is shown by σnoise. Color scale in the firing field plots ranges from 0 Hz (dark blue) to the maximal firing rate for each of the firing fields (dark red). DOI: http://dx.doi.org/10.7554/eLife.06444.036 [file elife06444s001.pdf]

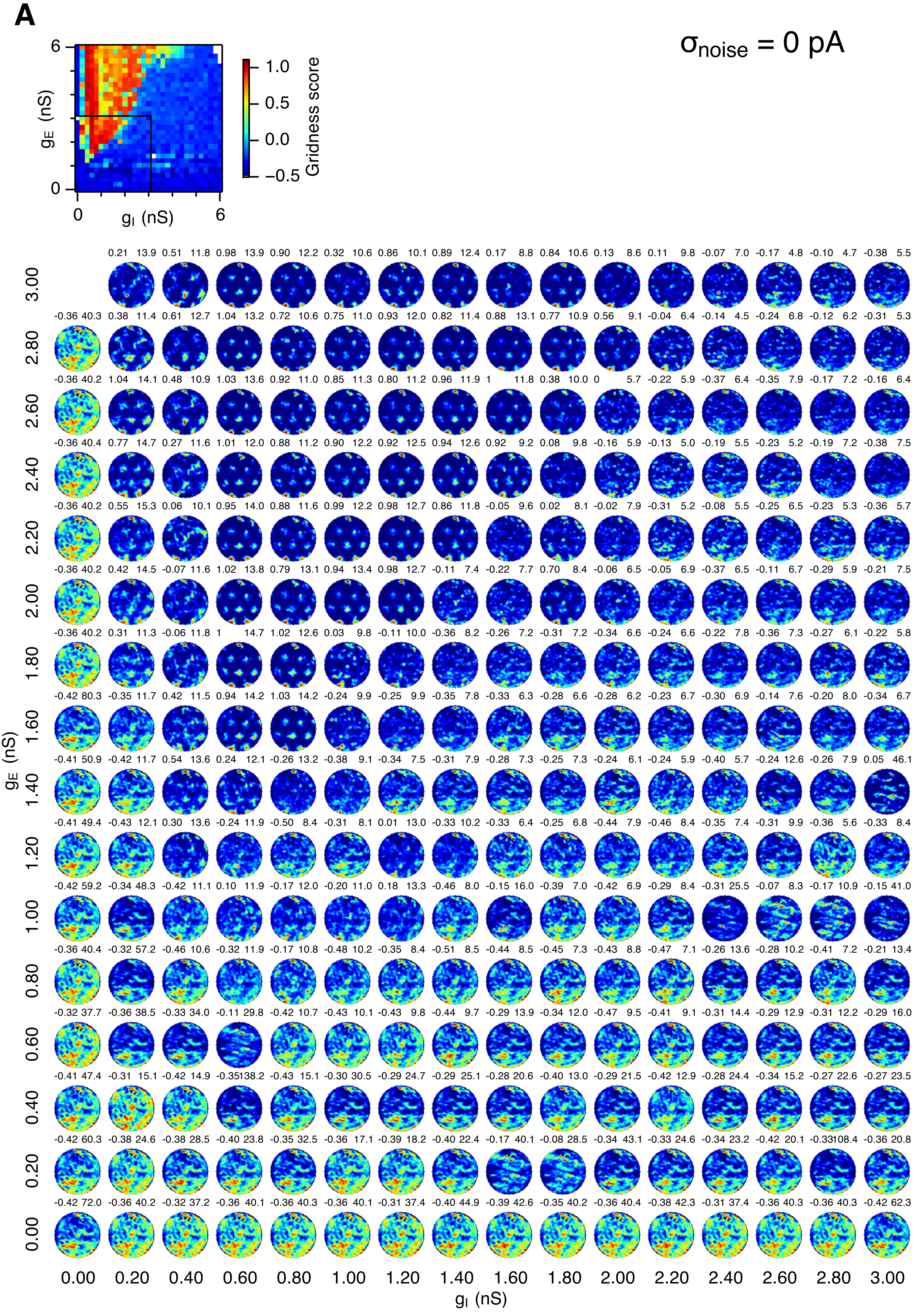

A
σnoise = 0 pA
6
0
gE (nS)
0
gI (nS)
6
1.0
0.5
0.0
−0.5
Gridness score
3.00: 0.21 13.9 | 0.51 11.8 | 0.98 13.9 | 0.90 12.2 | 0.32 10.6 | 0.86 10.1 | 0.89 12.4 | 0.17 8.8 | 0.84 10.6 | 0.13 8.6 | 0.11 9.8 | -0.07 7.0 | -0.17 4.8 | -0.10 4.7 | -0.38 5.5
2.80: -0.36 40.3 | 0.38 11.4 | 0.61 12.7 | 1.04 13.2 | 0.72 10.6 | 0.75 11.0 | 0.93 12.0 | 0.82 11.4 | 0.88 13.1 | 0.77 10.9 | 0.56 9.1 | -0.04 6.4 | -0.14 4.5 | -0.24 6.8 | -0.12 6.2 | -0.31 5.3
2.60: -0.36 40.2 | 1.04 14.1 | 0.48 10.9 | 1.03 13.6 | 0.92 11.0 | 0.85 11.3 | 0.80 11.2 | 0.96 11.9 | 1 11.8 | 0.38 10.0 | 0 5.7 | -0.22 5.9 | -0.37 6.4 | -0.35 7.9 | -0.17 7.2 | -0.16 6.4
2.40: -0.36 40.4 | 0.77 14.7 | 0.27 11.6 | 1.01 12.0 | 0.88 11.2 | 0.90 12.2 | 0.92 12.5 | 0.94 12.6 | 0.92 9.2 | 0.08 9.8 | -0.16 5.9 | -0.13 5.0 | -0.19 5.5 | -0.23 5.2 | -0.19 7.2 | -0.38 7.5
2.20: -0.36 40.2 | 0.55 15.3 | 0.06 10.1 | 0.95 14.0 | 0.88 11.6 | 0.99 12.2 | 0.98 12.7 | 0.86 11.8 | -0.05 9.6 | 0.02 8.1 | -0.02 7.9 | -0.31 5.2 | -0.08 5.5 | -0.25 6.5 | -0.23 5.3 | -0.36 5.7
2.00: -0.36 40.2 | 0.42 14.5 | -0.07 11.6 | 1.02 13.8 | 0.79 13.1 | 0.94 13.4 | 0.98 12.7 | -0.11 7.4 | -0.22 7.7 | 0.70 8.4 | -0.06 6.5 | -0.05 6.9 | -0.37 6.5 | -0.11 6.7 | -0.29 5.9 | -0.21 7.5
1.80: -0.36 40.2 | 0.31 11.3 | -0.06 11.8 | 1 14.7 | 1.02 12.6 | 0.03 9.8 | -0.11 10.0 | -0.36 8.2 | -0.26 7.2 | -0.31 7.2 | -0.34 6.6 | -0.24 6.6 | -0.22 7.8 | -0.36 7.3 | -0.27 6.1 | -0.22 5.8
1.60: -0.42 80.3 | -0.35 11.7 | 0.42 11.5 | 0.94 14.2 | 1.03 14.2 | -0.24 9.9 | -0.25 9.9 | -0.35 7.8 | -0.33 6.3 | -0.28 6.6 | -0.28 6.2 | -0.23 6.7 | -0.30 6.9 | -0.14 7.6 | -0.20 8.0 | -0.34 6.7
1.40: -0.41 50.9 | -0.42 11.7 | 0.54 13.6 | 0.24 12.1 | -0.26 13.2 | -0.38 9.1 | -0.34 7.5 | -0.31 7.9 | -0.28 7.3 | -0.25 7.3 | -0.24 6.1 | -0.24 5.9 | -0.40 5.7 | -0.24 12.6 | -0.26 7.9 | 0.05 46.1
1.20: -0.41 49.4 | -0.43 12.1 | 0.30 13.6 | -0.24 11.9 | -0.50 8.4 | -0.31 8.1 | 0.01 13.0 | -0.33 10.2 | -0.33 6.4 | -0.25 6.8 | -0.44 7.9 | -0.46 8.4 | -0.35 7.4 | -0.31 9.9 | -0.36 5.6 | -0.33 8.4
1.00: -0.42 59.2 | -0.34 48.3 | -0.42 11.1 | 0.10 11.9 | -0.17 12.0 | -0.20 11.0 | 0.18 13.3 | -0.46 8.0 | -0.15 16.0 | -0.39 7.0 | -0.42 6.9 | -0.29 8.4 | -0.31 25.5 | -0.07 8.3 | -0.17 10.9 | -0.15 41.0
0.80: -0.36 40.4 | -0.32 57.2 | -0.46 10.6 | -0.32 11.9 | -0.17 10.8 | -0.48 10.2 | -0.35 8.4 | -0.51 8.5 | -0.44 8.5 | -0.45 7.3 | -0.43 8.8 | -0.47 7.1 | -0.26 13.6 | -0.28 10.2 | -0.41 7.2 | -0.21 13.4
0.60: -0.32 37.7 | -0.36 38.5 | -0.33 34.0 | -0.11 29.8 | -0.42 10.7 | -0.43 10.1 | -0.43 9.8 | -0.44 9.7 | -0.29 13.9 | -0.34 12.0 | -0.47 9.5 | -0.41 9.1 | -0.31 14.4 | -0.29 12.9 | -0.31 12.2 | -0.29 16.0
0.40: -0.41 47.4 | -0.31 15.1 | -0.42 14.9 | -0.35138.2 | -0.43 15.1 | -0.30 30.5 | -0.29 24.7 | -0.29 25.1 | -0.28 20.6 | -0.40 13.0 | -0.29 21.5 | -0.42 12.9 | -0.28 24.4 | -0.34 15.2 | -0.27 22.6 | -0.27 23.5
0.20: -0.42 60.3 | -0.38 24.6 | -0.38 28.5 | -0.40 23.8 | -0.35 32.5 | -0.36 17.1 | -0.39 18.2 | -0.40 22.4 | -0.17 40.1 | -0.08 28.5 | -0.34 43.1 | -0.33 24.6 | -0.34 23.2 | -0.42 20.1 | -0.33108.4 | -0.36 20.8
0.00: -0.42 72.0 | -0.36 40.2 | -0.32 37.2 | -0.36 40.1 | -0.36 40.3 | -0.36 40.1 | -0.31 37.4 | -0.40 44.9 | -0.39 42.6 | -0.35 40.2 | -0.36 40.4 | -0.38 42.3 | -0.31 37.4 | -0.36 40.3 | -0.36 40.3 | -0.42 62.3
gE (nS)
0.00 0.20 0.40 0.60 0.80 1.00 1.20 1.40 1.60 1.80 2.00 2.20 2.40 2.60 2.80 3.00
gI (nS)

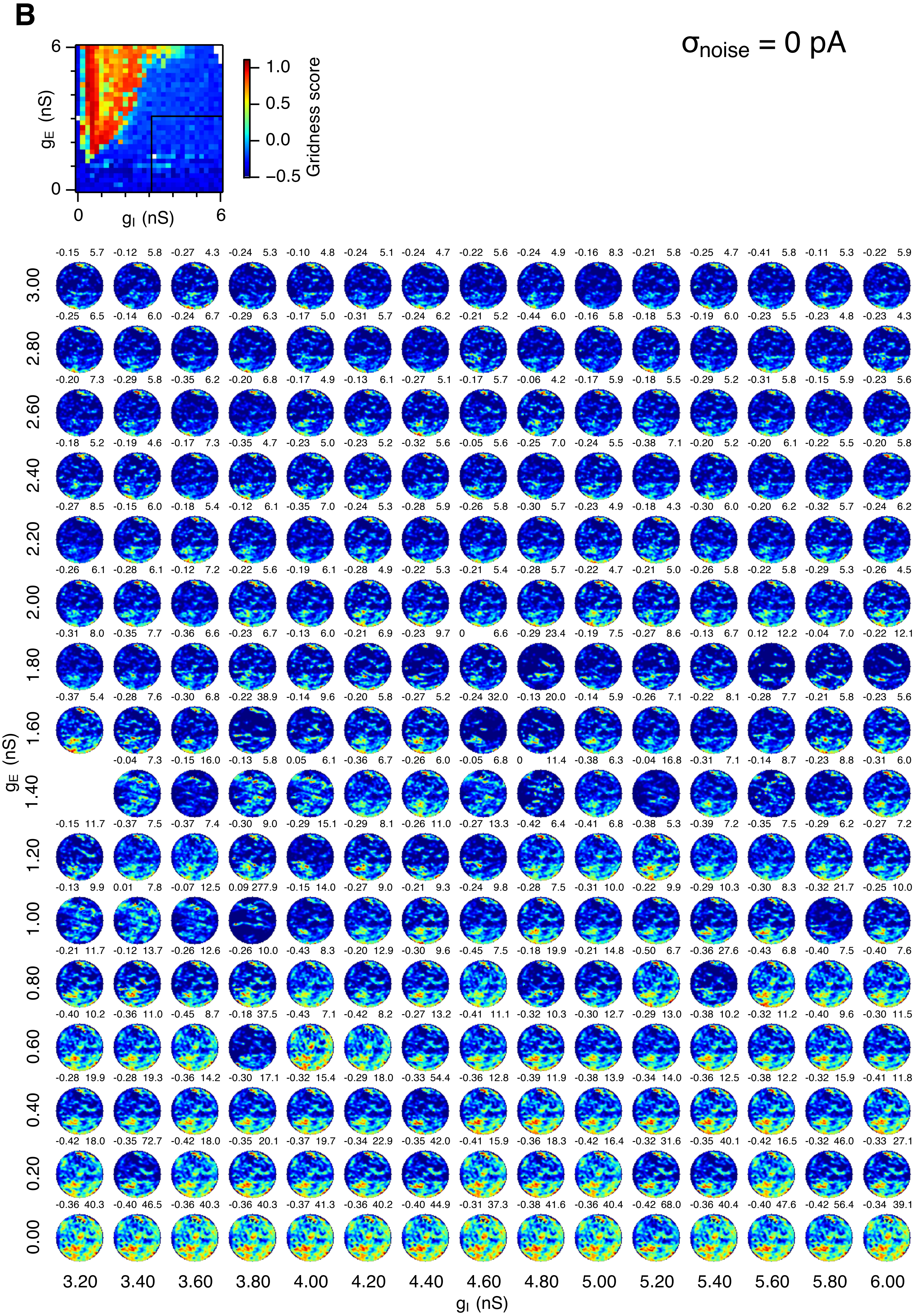

B
σnoise = 0 pA
Gridness score
1.0
0.5
0.0
−0.5
gE (nS)
gI (nS)
0
6
-0.15 5.7 -0.12 5.8 -0.27 4.3 -0.24 5.3 -0.10 4.8 -0.24 5.1 -0.24 4.7 -0.22 5.6 -0.24 4.9 -0.16 8.3 -0.21 5.8 -0.25 4.7 -0.41 5.8 -0.11 5.3 -0.22 5.9
3.00
-0.25 6.5 -0.14 6.0 -0.24 6.7 -0.29 6.3 -0.17 5.0 -0.31 5.7 -0.24 6.2 -0.21 5.2 -0.44 6.0 -0.16 5.8 -0.18 5.3 -0.19 6.0 -0.23 5.5 -0.23 4.8 -0.23 4.3
2.80
-0.20 7.3 -0.29 5.8 -0.35 6.2 -0.20 6.8 -0.17 4.9 -0.13 6.1 -0.27 5.1 -0.17 5.7 -0.06 4.2 -0.17 5.9 -0.18 5.5 -0.29 5.2 -0.31 5.8 -0.15 5.9 -0.23 5.6
2.60
-0.18 5.2 -0.19 4.6 -0.17 7.3 -0.35 4.7 -0.23 5.0 -0.23 5.2 -0.32 5.6 -0.05 5.6 -0.25 7.0 -0.24 5.5 -0.38 7.1 -0.20 5.2 -0.20 6.1 -0.22 5.5 -0.20 5.8
2.40
-0.27 8.5 -0.15 6.0 -0.18 5.4 -0.12 6.1 -0.35 7.0 -0.24 5.3 -0.28 5.9 -0.26 5.8 -0.30 5.7 -0.23 4.9 -0.18 4.3 -0.30 6.0 -0.20 6.2 -0.32 5.7 -0.24 6.2
2.20
-0.26 6.1 -0.28 6.1 -0.12 7.2 -0.22 5.6 -0.19 6.1 -0.28 4.9 -0.22 5.3 -0.21 5.4 -0.28 5.7 -0.22 4.7 -0.21 5.0 -0.26 5.8 -0.22 5.8 -0.29 5.3 -0.26 4.5
2.00
-0.31 8.0 -0.35 7.7 -0.36 6.6 -0.23 6.7 -0.13 6.0 -0.21 6.9 -0.23 9.7 0 6.6 -0.29 23.4 -0.19 7.5 -0.27 8.6 -0.13 6.7 0.12 12.2 -0.04 7.0 -0.22 12.1
1.80
-0.37 5.4 -0.28 7.6 -0.30 6.8 -0.22 38.9 -0.14 9.6 -0.20 5.8 -0.27 5.2 -0.24 32.0 -0.13 20.0 -0.14 5.9 -0.26 7.1 -0.22 8.1 -0.28 7.7 -0.21 5.8 -0.23 5.6
1.60
-0.04 7.3 -0.15 16.0 -0.13 5.8 0.05 6.1 -0.36 6.7 -0.26 6.0 -0.05 6.8 0 11.4 -0.38 6.3 -0.04 16.8 -0.31 7.1 -0.14 8.7 -0.23 8.8 -0.31 6.0
1.40
-0.15 11.7 -0.37 7.5 -0.37 7.4 -0.30 9.0 -0.29 15.1 -0.29 8.1 -0.26 11.0 -0.27 13.3 -0.42 6.4 -0.41 6.8 -0.38 5.3 -0.39 7.2 -0.35 7.5 -0.29 6.2 -0.27 7.2
1.20
-0.13 9.9 0.01 7.8 -0.07 12.5 0.09 277.9 -0.15 14.0 -0.27 9.0 -0.21 9.3 -0.24 9.8 -0.28 7.5 -0.31 10.0 -0.22 9.9 -0.29 10.3 -0.30 8.3 -0.32 21.7 -0.25 10.0
1.00
-0.21 11.7 -0.12 13.7 -0.26 12.6 -0.26 10.0 -0.43 8.3 -0.20 12.9 -0.30 9.6 -0.45 7.5 -0.18 19.9 -0.21 14.8 -0.50 6.7 -0.36 27.6 -0.43 6.8 -0.40 7.5 -0.40 7.6
0.80
-0.40 10.2 -0.36 11.0 -0.45 8.7 -0.18 37.5 -0.43 7.1 -0.42 8.2 -0.27 13.2 -0.41 11.1 -0.32 10.3 -0.30 12.7 -0.29 13.0 -0.38 10.2 -0.32 11.2 -0.40 9.6 -0.30 11.5
0.60
-0.28 19.9 -0.28 19.3 -0.36 14.2 -0.30 17.1 -0.32 15.4 -0.29 18.0 -0.33 54.4 -0.36 12.8 -0.39 11.9 -0.38 13.9 -0.34 14.0 -0.36 12.5 -0.38 12.2 -0.32 15.9 -0.41 11.8
0.40
-0.42 18.0 -0.35 72.7 -0.42 18.0 -0.35 20.1 -0.37 19.7 -0.34 22.9 -0.35 42.0 -0.41 15.9 -0.36 18.3 -0.42 16.4 -0.32 31.6 -0.35 40.1 -0.42 16.5 -0.32 46.0 -0.33 27.1
0.20
-0.36 40.3 -0.40 46.5 -0.36 40.3 -0.36 40.3 -0.37 41.3 -0.36 40.2 -0.40 44.9 -0.31 37.3 -0.38 41.6 -0.36 40.4 -0.42 68.0 -0.36 40.4 -0.40 47.6 -0.42 56.4 -0.34 39.1
0.00
gE (nS)
3.20 3.40 3.60 3.80 4.00 4.20 4.40 4.60 4.80 5.00 5.20 5.40 5.60 5.80 6.00
gI (nS)

C

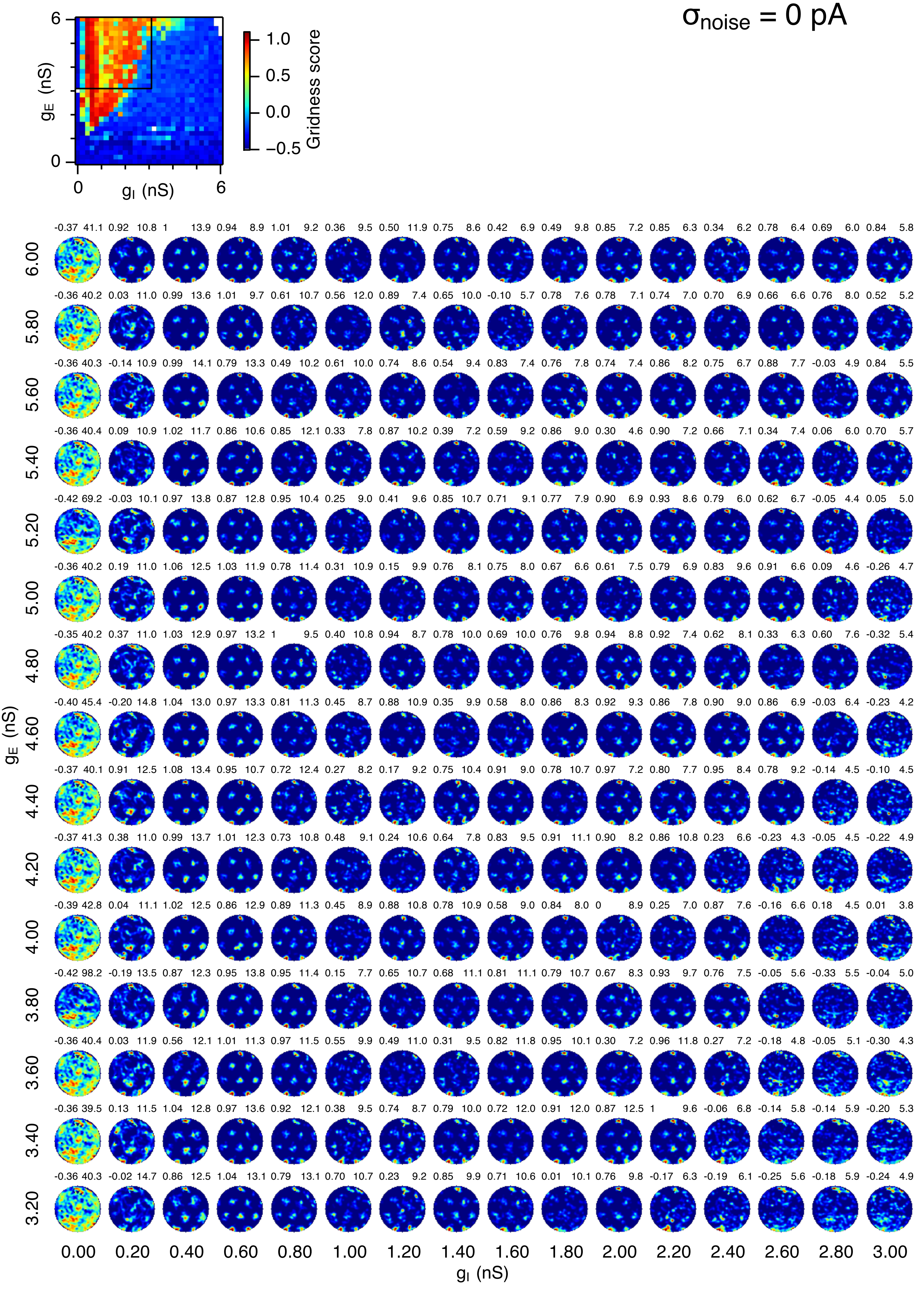

σ_{noise} = 0 pA
g_E (nS)
g_I (nS)
0
6
Gridness score
1.0
0.5
0.0
−0.5
6.00: -0.37 41.1 | 0.92 10.8 | 1 13.9 | 0.94 8.9 | 1.01 9.2 | 0.36 9.5 | 0.50 11.9 | 0.75 8.6 | 0.42 6.9 | 0.49 9.8 | 0.85 7.2 | 0.85 6.3 | 0.34 6.2 | 0.78 6.4 | 0.69 6.0 | 0.84 5.8
5.80: -0.36 40.2 | 0.03 11.0 | 0.99 13.6 | 1.01 9.7 | 0.61 10.7 | 0.56 12.0 | 0.89 7.4 | 0.65 10.0 | -0.10 5.7 | 0.78 7.6 | 0.78 7.1 | 0.74 7.0 | 0.70 6.9 | 0.66 6.6 | 0.76 8.0 | 0.52 5.2
5.60: -0.36 40.3 | -0.14 10.9 | 0.99 14.1 | 0.79 13.3 | 0.49 10.2 | 0.61 10.0 | 0.74 8.6 | 0.54 9.4 | 0.83 7.4 | 0.76 7.8 | 0.74 7.4 | 0.86 8.2 | 0.75 6.7 | 0.88 7.7 | -0.03 4.9 | 0.84 5.5
5.40: -0.36 40.4 | 0.09 10.9 | 1.02 11.7 | 0.86 10.6 | 0.85 12.1 | 0.33 7.8 | 0.87 10.2 | 0.39 7.2 | 0.59 9.2 | 0.86 9.0 | 0.30 4.6 | 0.90 7.2 | 0.66 7.1 | 0.34 7.4 | 0.06 6.0 | 0.70 5.7
5.20: -0.42 69.2 | -0.03 10.1 | 0.97 13.8 | 0.87 12.8 | 0.95 10.4 | 0.25 9.0 | 0.41 9.6 | 0.85 10.7 | 0.71 9.1 | 0.77 7.9 | 0.90 6.9 | 0.93 8.6 | 0.79 6.0 | 0.62 6.7 | -0.05 4.4 | 0.05 5.0
5.00: -0.36 40.2 | 0.19 11.0 | 1.06 12.5 | 1.03 11.9 | 0.78 11.4 | 0.31 10.9 | 0.15 9.9 | 0.76 8.1 | 0.75 8.0 | 0.67 6.6 | 0.61 7.5 | 0.79 6.9 | 0.83 9.6 | 0.91 6.6 | 0.09 4.6 | -0.26 4.7
4.80: -0.35 40.2 | 0.37 11.0 | 1.03 12.9 | 0.97 13.2 | 1 9.5 | 0.40 10.8 | 0.94 8.7 | 0.78 10.0 | 0.69 10.0 | 0.76 9.8 | 0.94 8.8 | 0.92 7.4 | 0.62 8.1 | 0.33 6.3 | 0.60 7.6 | -0.32 5.4
4.60: -0.40 45.4 | -0.20 14.8 | 1.04 13.0 | 0.97 13.3 | 0.81 11.3 | 0.45 8.7 | 0.88 10.9 | 0.35 9.9 | 0.58 8.0 | 0.86 8.3 | 0.92 9.3 | 0.86 7.8 | 0.90 9.0 | 0.86 6.9 | -0.03 6.4 | -0.23 4.2
4.40: -0.37 40.1 | 0.91 12.5 | 1.08 13.4 | 0.95 10.7 | 0.72 12.4 | 0.27 8.2 | 0.17 9.2 | 0.75 10.4 | 0.91 9.0 | 0.78 10.7 | 0.97 7.2 | 0.80 7.7 | 0.95 8.4 | 0.78 9.2 | -0.14 4.5 | -0.10 4.5
4.20: -0.37 41.3 | 0.38 11.0 | 0.99 13.7 | 1.01 12.3 | 0.73 10.8 | 0.48 9.1 | 0.24 10.6 | 0.64 7.8 | 0.83 9.5 | 0.91 11.1 | 0.90 8.2 | 0.86 10.8 | 0.23 6.6 | -0.23 4.3 | -0.05 4.5 | -0.22 4.9
4.00: -0.39 42.8 | 0.04 11.1 | 1.02 12.5 | 0.86 12.9 | 0.89 11.3 | 0.45 8.9 | 0.88 10.8 | 0.78 10.9 | 0.58 9.0 | 0.84 8.0 | 0 8.9 | 0.25 7.0 | 0.87 7.6 | -0.16 6.6 | 0.18 4.5 | 0.01 3.8
3.80: -0.42 98.2 | -0.19 13.5 | 0.87 12.3 | 0.95 13.8 | 0.95 11.4 | 0.15 7.7 | 0.65 10.7 | 0.68 11.1 | 0.81 11.1 | 0.79 10.7 | 0.67 8.3 | 0.93 9.7 | 0.76 7.5 | -0.05 5.6 | -0.33 5.5 | -0.04 5.0
3.60: -0.36 40.4 | 0.03 11.9 | 0.56 12.1 | 1.01 11.3 | 0.97 11.5 | 0.55 9.9 | 0.49 11.0 | 0.31 9.5 | 0.82 11.8 | 0.95 10.1 | 0.30 7.2 | 0.96 11.8 | 0.27 7.2 | -0.18 4.8 | -0.05 5.1 | -0.30 4.3
3.40: -0.36 39.5 | 0.13 11.5 | 1.04 12.8 | 0.97 13.6 | 0.92 12.1 | 0.38 9.5 | 0.74 8.7 | 0.79 10.0 | 0.72 12.0 | 0.91 12.0 | 0.87 12.5 | 1 9.6 | -0.06 6.8 | -0.14 5.8 | -0.14 5.9 | -0.20 5.3
3.20: -0.36 40.3 | -0.02 14.7 | 0.86 12.5 | 1.04 13.1 | 0.79 13.1 | 0.70 10.7 | 0.23 9.2 | 0.85 9.9 | 0.71 10.6 | 0.01 10.1 | 0.76 9.8 | -0.17 6.3 | -0.19 6.1 | -0.25 5.6 | -0.18 5.9 | -0.24 4.9
0.00 0.20 0.40 0.60 0.80 1.00 1.20 1.40 1.60 1.80 2.00 2.20 2.40 2.60 2.80 3.00

D

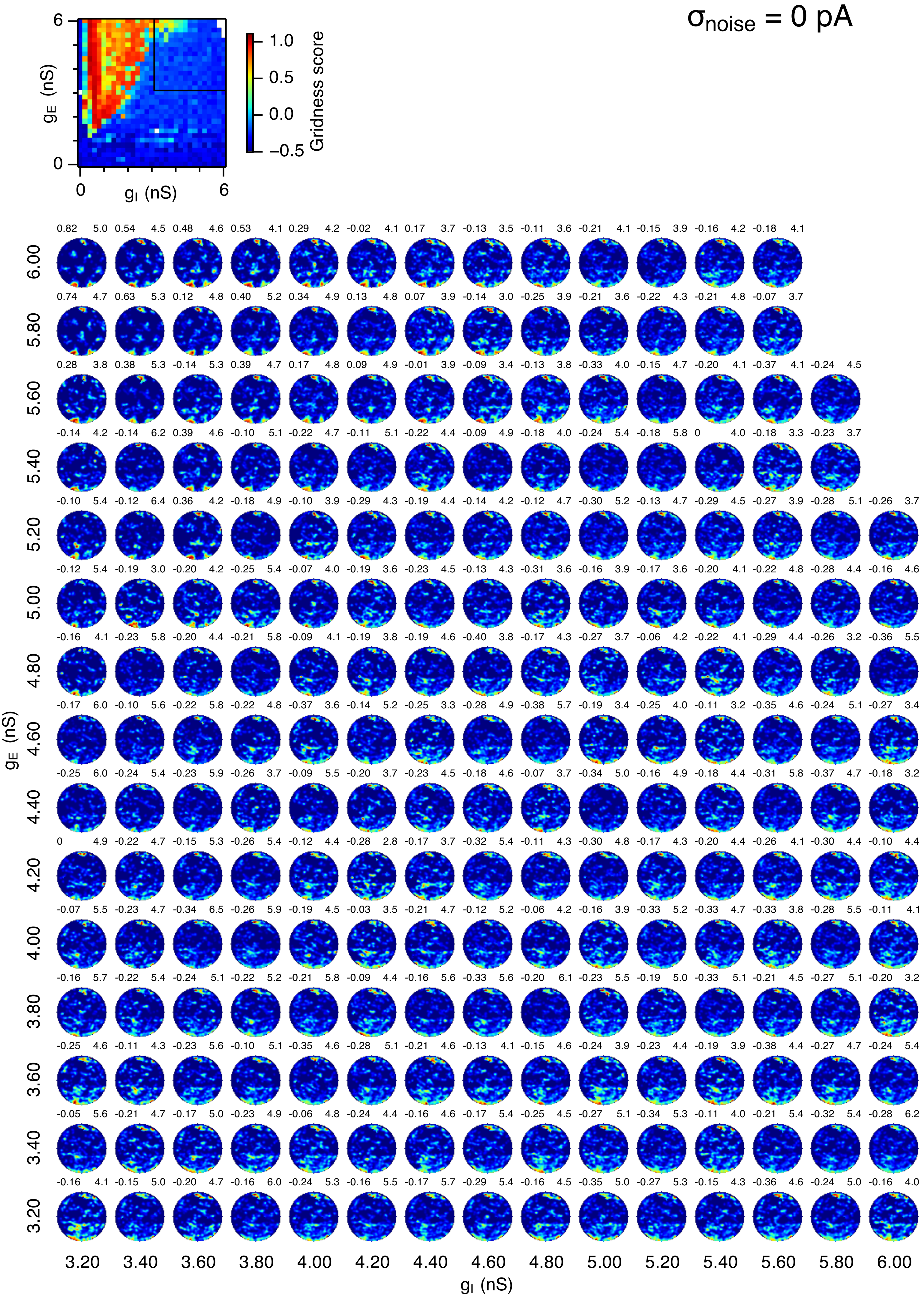

σ_{noise} = 0 pA
6
g_E (nS)
0
0
g_I (nS)
6
1.0
0.5
0.0
−0.5
Gridness score
6.00: 0.82 5.0 | 0.54 4.5 | 0.48 4.6 | 0.53 4.1 | 0.29 4.2 | -0.02 4.1 | 0.17 3.7 | -0.13 3.5 | -0.11 3.6 | -0.21 4.1 | -0.15 3.9 | -0.16 4.2 | -0.18 4.1
5.80: 0.74 4.7 | 0.63 5.3 | 0.12 4.8 | 0.40 5.2 | 0.34 4.9 | 0.13 4.8 | 0.07 3.9 | -0.14 3.0 | -0.25 3.9 | -0.21 3.6 | -0.22 4.3 | -0.21 4.8 | -0.07 3.7
5.60: 0.28 3.8 | 0.38 5.3 | -0.14 5.3 | 0.39 4.7 | 0.17 4.8 | 0.09 4.9 | -0.01 3.9 | -0.09 3.4 | -0.13 3.8 | -0.33 4.0 | -0.15 4.7 | -0.20 4.1 | -0.37 4.1 | -0.24 4.5
5.40: -0.14 4.2 | -0.14 6.2 | 0.39 4.6 | -0.10 5.1 | -0.22 4.7 | -0.11 5.1 | -0.22 4.4 | -0.09 4.9 | -0.18 4.0 | -0.24 5.4 | -0.18 5.8 | 0 4.0 | -0.18 3.3 | -0.23 3.7
5.20: -0.10 5.4 | -0.12 6.4 | 0.36 4.2 | -0.18 4.9 | -0.10 3.9 | -0.29 4.3 | -0.19 4.4 | -0.14 4.2 | -0.12 4.7 | -0.30 5.2 | -0.13 4.7 | -0.29 4.5 | -0.27 3.9 | -0.28 5.1 | -0.26 3.7
5.00: -0.12 5.4 | -0.19 3.0 | -0.20 4.2 | -0.25 5.4 | -0.07 4.0 | -0.19 3.6 | -0.23 4.5 | -0.13 4.3 | -0.31 3.6 | -0.16 3.9 | -0.17 3.6 | -0.20 4.1 | -0.22 4.8 | -0.28 4.4 | -0.16 4.6
4.80: -0.16 4.1 | -0.23 5.8 | -0.20 4.4 | -0.21 5.8 | -0.09 4.1 | -0.19 3.8 | -0.19 4.6 | -0.40 3.8 | -0.17 4.3 | -0.27 3.7 | -0.06 4.2 | -0.22 4.1 | -0.29 4.4 | -0.26 3.2 | -0.36 5.5
4.60: -0.17 6.0 | -0.10 5.6 | -0.22 5.8 | -0.22 4.8 | -0.37 3.6 | -0.14 5.2 | -0.25 3.3 | -0.28 4.9 | -0.38 5.7 | -0.19 3.4 | -0.25 4.0 | -0.11 3.2 | -0.35 4.6 | -0.24 5.1 | -0.27 3.4
4.40: -0.25 6.0 | -0.24 5.4 | -0.23 5.9 | -0.26 3.7 | -0.09 5.5 | -0.20 3.7 | -0.23 4.5 | -0.18 4.6 | -0.07 3.7 | -0.34 5.0 | -0.16 4.9 | -0.18 4.4 | -0.31 5.8 | -0.37 4.7 | -0.18 3.2
4.20: 0 4.9 | -0.22 4.7 | -0.15 5.3 | -0.26 5.4 | -0.12 4.4 | -0.28 2.8 | -0.17 3.7 | -0.32 5.4 | -0.11 4.3 | -0.30 4.8 | -0.17 4.3 | -0.20 4.4 | -0.26 4.1 | -0.30 4.4 | -0.10 4.4
4.00: -0.07 5.5 | -0.23 4.7 | -0.34 6.5 | -0.26 5.9 | -0.19 4.5 | -0.03 3.5 | -0.21 4.7 | -0.12 5.2 | -0.06 4.2 | -0.16 3.9 | -0.33 5.2 | -0.33 4.7 | -0.33 3.8 | -0.28 5.5 | -0.11 4.1
3.80: -0.16 5.7 | -0.22 5.4 | -0.24 5.1 | -0.22 5.2 | -0.21 5.8 | -0.09 4.4 | -0.16 5.6 | -0.33 5.6 | -0.20 6.1 | -0.23 5.5 | -0.19 5.0 | -0.33 5.1 | -0.21 4.5 | -0.27 5.1 | -0.20 3.2
3.60: -0.25 4.6 | -0.11 4.3 | -0.23 5.6 | -0.10 5.1 | -0.35 4.6 | -0.28 5.1 | -0.21 4.6 | -0.13 4.1 | -0.15 4.6 | -0.24 3.9 | -0.23 4.4 | -0.19 3.9 | -0.38 4.4 | -0.27 4.7 | -0.24 5.4
3.40: -0.05 5.6 | -0.21 4.7 | -0.17 5.0 | -0.23 4.9 | -0.06 4.8 | -0.24 4.4 | -0.16 4.6 | -0.17 5.4 | -0.25 4.5 | -0.27 5.1 | -0.34 5.3 | -0.11 4.0 | -0.21 5.4 | -0.32 5.4 | -0.28 6.2
3.20: -0.16 4.1 | -0.15 5.0 | -0.20 4.7 | -0.16 6.0 | -0.24 5.3 | -0.16 5.5 | -0.17 5.7 | -0.29 5.4 | -0.16 4.5 | -0.35 5.0 | -0.27 5.3 | -0.15 4.3 | -0.36 4.6 | -0.24 5.0 | -0.16 4.0
g_E (nS)
3.20 3.40 3.60 3.80 4.00 4.20 4.40 4.60 4.80 5.00 5.20 5.40 5.60 5.80 6.00
g_I (nS)

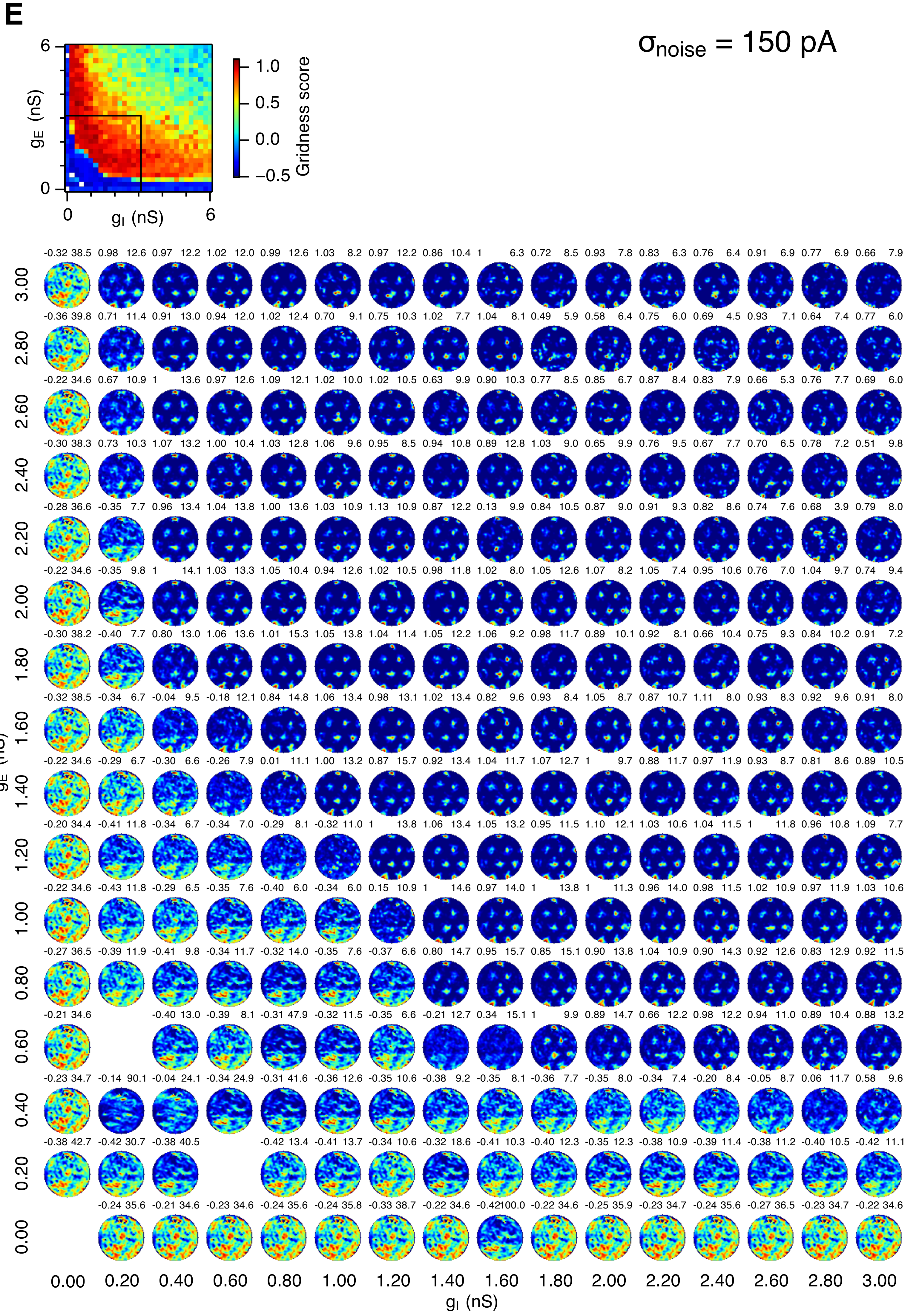
E
σnoise = 150 pA
gE (nS)
gI (nS)
Gridness score
1.0
0.5
0.0
−0.5
6
0
-0.32 38.5 0.98 12.6 0.97 12.2 1.02 12.0 0.99 12.6 1.03 8.2 0.97 12.2 0.86 10.4 1 6.3 0.72 8.5 0.93 7.8 0.83 6.3 0.76 6.4 0.91 6.9 0.77 6.9 0.66 7.9
-0.36 39.8 0.71 11.4 0.91 13.0 0.94 12.0 1.02 12.4 0.70 9.1 0.75 10.3 1.02 7.7 1.04 8.1 0.49 5.9 0.58 6.4 0.75 6.0 0.69 4.5 0.93 7.1 0.64 7.4 0.77 6.0
-0.22 34.6 0.67 10.9 1 13.6 0.97 12.6 1.09 12.1 1.02 10.0 1.02 10.5 0.63 9.9 0.90 10.3 0.77 8.5 0.85 6.7 0.87 8.4 0.83 7.9 0.66 5.3 0.76 7.7 0.69 6.0
-0.30 38.3 0.73 10.3 1.07 13.2 1.00 10.4 1.03 12.8 1.06 9.6 0.95 8.5 0.94 10.8 0.89 12.8 1.03 9.0 0.65 9.9 0.76 9.5 0.67 7.7 0.70 6.5 0.78 7.2 0.51 9.8
-0.28 36.6 -0.35 7.7 0.96 13.4 1.04 13.8 1.00 13.6 1.03 10.9 1.13 10.9 0.87 12.2 0.13 9.9 0.84 10.5 0.87 9.0 0.91 9.3 0.82 8.6 0.74 7.6 0.68 3.9 0.79 8.0
-0.22 34.6 -0.35 9.8 1 14.1 1.03 13.3 1.05 10.4 0.94 12.6 1.02 10.5 0.98 11.8 1.02 8.0 1.05 12.6 1.07 8.2 1.05 7.4 0.95 10.6 0.76 7.0 1.04 9.7 0.74 9.4
-0.30 38.2 -0.40 7.7 0.80 13.0 1.06 13.6 1.01 15.3 1.05 13.8 1.04 11.4 1.05 12.2 1.06 9.2 0.98 11.7 0.89 10.1 0.92 8.1 0.66 10.4 0.75 9.3 0.84 10.2 0.91 7.2
-0.32 38.5 -0.34 6.7 -0.04 9.5 -0.18 12.1 0.84 14.8 1.06 13.4 0.98 13.1 1.02 13.4 0.82 9.6 0.93 8.4 1.05 8.7 0.87 10.7 1.11 8.0 0.93 8.3 0.92 9.6 0.91 8.0
-0.22 34.6 -0.29 6.7 -0.30 6.6 -0.26 7.9 0.01 11.1 1.00 13.2 0.87 15.7 0.92 13.4 1.04 11.7 1.07 12.7 1 9.7 0.88 11.7 0.97 11.9 0.93 8.7 0.81 8.6 0.89 10.5
-0.20 34.4 -0.41 11.8 -0.34 6.7 -0.34 7.0 -0.29 8.1 -0.32 11.0 1 13.8 1.06 13.4 1.05 13.2 0.95 11.5 1.10 12.1 1.03 10.6 1.04 11.5 1 11.8 0.96 10.8 1.09 7.7
-0.22 34.6 -0.43 11.8 -0.29 6.5 -0.35 7.6 -0.40 6.0 -0.34 6.0 0.15 10.9 1 14.6 0.97 14.0 1 13.8 1 11.3 0.96 14.0 0.98 11.5 1.02 10.9 0.97 11.9 1.03 10.6
-0.27 36.5 -0.39 11.9 -0.41 9.8 -0.34 11.7 -0.32 14.0 -0.35 7.6 -0.37 6.6 0.80 14.7 0.95 15.7 0.85 15.1 0.90 13.8 1.04 10.9 0.90 14.3 0.92 12.6 0.83 12.9 0.92 11.5
-0.21 34.6 -0.40 13.0 -0.39 8.1 -0.31 47.9 -0.32 11.5 -0.35 6.6 -0.21 12.7 0.34 15.1 1 9.9 0.89 14.7 0.66 12.2 0.98 12.2 0.94 11.0 0.89 10.4 0.88 13.2
-0.23 34.7 -0.14 90.1 -0.04 24.1 -0.34 24.9 -0.31 41.6 -0.36 12.6 -0.35 10.6 -0.38 9.2 -0.35 8.1 -0.36 7.7 -0.35 8.0 -0.34 7.4 -0.20 8.4 -0.05 8.7 0.06 11.7 0.58 9.6
-0.38 42.7 -0.42 30.7 -0.38 40.5 -0.42 13.4 -0.41 13.7 -0.34 10.6 -0.32 18.6 -0.41 10.3 -0.40 12.3 -0.35 12.3 -0.38 10.9 -0.39 11.4 -0.38 11.2 -0.40 10.5 -0.42 11.1
-0.24 35.6 -0.21 34.6 -0.23 34.6 -0.24 35.6 -0.24 35.8 -0.33 38.7 -0.22 34.6 -0.42100.0 -0.22 34.6 -0.25 35.9 -0.23 34.7 -0.24 35.6 -0.27 36.5 -0.23 34.7 -0.22 34.6
3.00
2.80
2.60
2.40
2.20
2.00
1.80
1.60
1.40
1.20
1.00
0.80
0.60
0.40
0.20
0.00
0.00 0.20 0.40 0.60 0.80 1.00 1.20 1.40 1.60 1.80 2.00 2.20 2.40 2.60 2.80 3.00

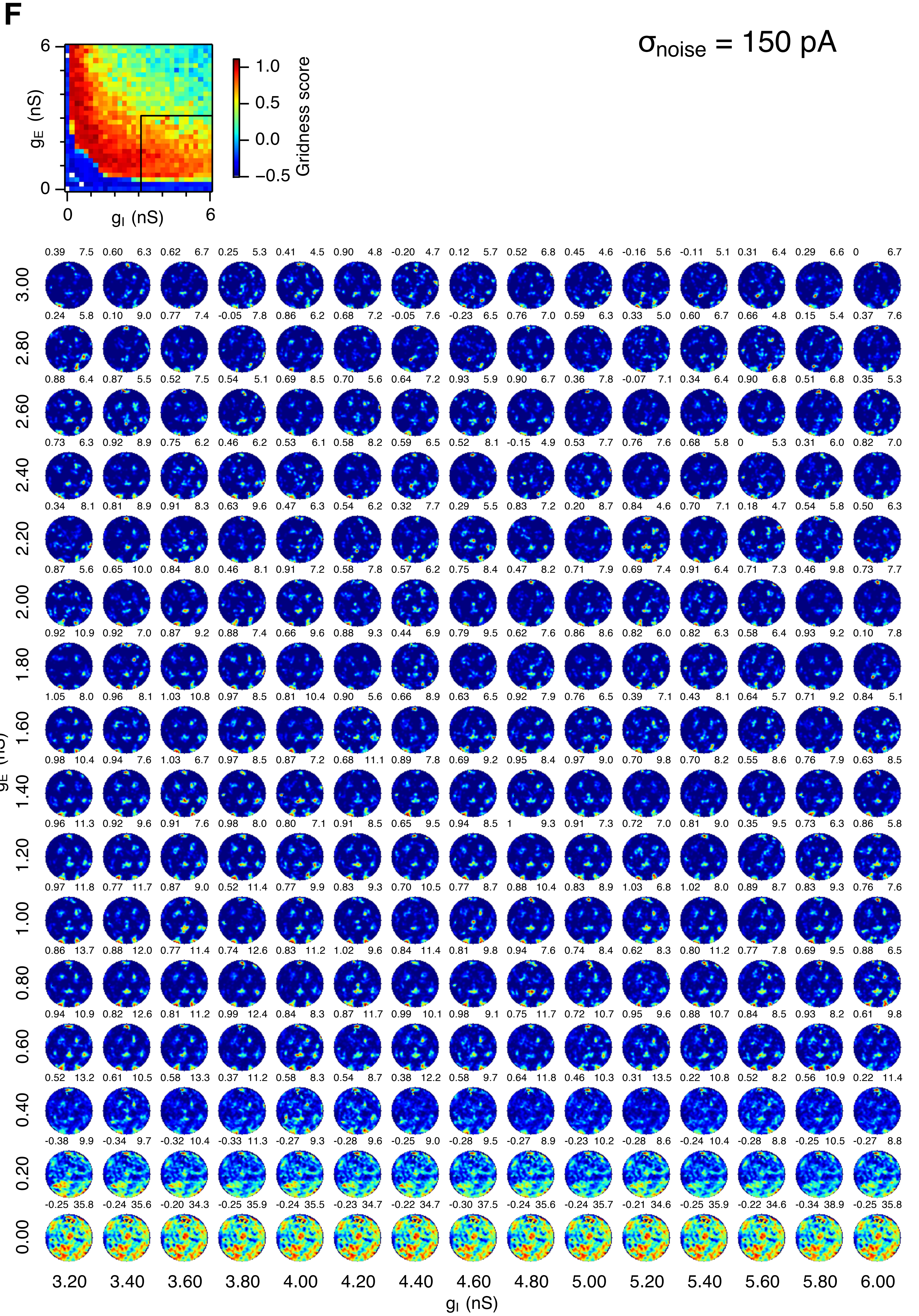
F
σnoise = 150 pA
6
gE (nS)
0
0
gI (nS)
6
1.0
0.5
0.0
−0.5
Gridness score
3.00: 0.39 7.5 | 0.60 6.3 | 0.62 6.7 | 0.25 5.3 | 0.41 4.5 | 0.90 4.8 | -0.20 4.7 | 0.12 5.7 | 0.52 6.8 | 0.45 4.6 | -0.16 5.6 | -0.11 5.1 | 0.31 6.4 | 0.29 6.6 | 0 6.7
2.80: 0.24 5.8 | 0.10 9.0 | 0.77 7.4 | -0.05 7.8 | 0.86 6.2 | 0.68 7.2 | -0.05 7.6 | -0.23 6.5 | 0.76 7.0 | 0.59 6.3 | 0.33 5.0 | 0.60 6.7 | 0.66 4.8 | 0.15 5.4 | 0.37 7.6
2.60: 0.88 6.4 | 0.87 5.5 | 0.52 7.5 | 0.54 5.1 | 0.69 8.5 | 0.70 5.6 | 0.64 7.2 | 0.93 5.9 | 0.90 6.7 | 0.36 7.8 | -0.07 7.1 | 0.34 6.4 | 0.90 6.8 | 0.51 6.8 | 0.35 5.3
2.40: 0.73 6.3 | 0.92 8.9 | 0.75 6.2 | 0.46 6.2 | 0.53 6.1 | 0.58 8.2 | 0.59 6.5 | 0.52 8.1 | -0.15 4.9 | 0.53 7.7 | 0.76 7.6 | 0.68 5.8 | 0 5.3 | 0.31 6.0 | 0.82 7.0
2.20: 0.34 8.1 | 0.81 8.9 | 0.91 8.3 | 0.63 9.6 | 0.47 6.3 | 0.54 6.2 | 0.32 7.7 | 0.29 5.5 | 0.83 7.2 | 0.20 8.7 | 0.84 4.6 | 0.70 7.1 | 0.18 4.7 | 0.54 5.8 | 0.50 6.3
2.00: 0.87 5.6 | 0.65 10.0 | 0.84 8.0 | 0.46 8.1 | 0.91 7.2 | 0.58 7.8 | 0.57 6.2 | 0.75 8.4 | 0.47 8.2 | 0.71 7.9 | 0.69 7.4 | 0.91 6.4 | 0.71 7.3 | 0.46 9.8 | 0.73 7.7
1.80: 0.92 10.9 | 0.92 7.0 | 0.87 9.2 | 0.88 7.4 | 0.66 9.6 | 0.88 9.3 | 0.44 6.9 | 0.79 9.5 | 0.62 7.6 | 0.86 8.6 | 0.82 6.0 | 0.82 6.3 | 0.58 6.4 | 0.93 9.2 | 0.10 7.8
1.60: 1.05 8.0 | 0.96 8.1 | 1.03 10.8 | 0.97 8.5 | 0.81 10.4 | 0.90 5.6 | 0.66 8.9 | 0.63 6.5 | 0.92 7.9 | 0.76 6.5 | 0.39 7.1 | 0.43 8.1 | 0.64 5.7 | 0.71 9.2 | 0.84 5.1
1.40: 0.98 10.4 | 0.94 7.6 | 1.03 6.7 | 0.97 8.5 | 0.87 7.2 | 0.68 11.1 | 0.89 7.8 | 0.69 9.2 | 0.95 8.4 | 0.97 9.0 | 0.70 9.8 | 0.70 8.2 | 0.55 8.6 | 0.76 7.9 | 0.63 8.5
1.20: 0.96 11.3 | 0.92 9.6 | 0.91 7.6 | 0.98 8.0 | 0.80 7.1 | 0.91 8.5 | 0.65 9.5 | 0.94 8.5 | 1 9.3 | 0.91 7.3 | 0.72 7.0 | 0.81 9.0 | 0.35 9.5 | 0.73 6.3 | 0.86 5.8
1.00: 0.97 11.8 | 0.77 11.7 | 0.87 9.0 | 0.52 11.4 | 0.77 9.9 | 0.83 9.3 | 0.70 10.5 | 0.77 8.7 | 0.88 10.4 | 0.83 8.9 | 1.03 6.8 | 1.02 8.0 | 0.89 8.7 | 0.83 9.3 | 0.76 7.6
0.80: 0.86 13.7 | 0.88 12.0 | 0.77 11.4 | 0.74 12.6 | 0.83 11.2 | 1.02 9.6 | 0.84 11.4 | 0.81 9.8 | 0.94 7.6 | 0.74 8.4 | 0.62 8.3 | 0.80 11.2 | 0.77 7.8 | 0.69 9.5 | 0.88 6.5
0.60: 0.94 10.9 | 0.82 12.6 | 0.81 11.2 | 0.99 12.4 | 0.84 8.3 | 0.87 11.7 | 0.99 10.1 | 0.98 9.1 | 0.75 11.7 | 0.72 10.7 | 0.95 9.6 | 0.88 10.7 | 0.84 8.5 | 0.93 8.2 | 0.61 9.8
0.40: 0.52 13.2 | 0.61 10.5 | 0.58 13.3 | 0.37 11.2 | 0.58 8.3 | 0.54 8.7 | 0.38 12.2 | 0.58 9.7 | 0.64 11.8 | 0.46 10.3 | 0.31 13.5 | 0.22 10.8 | 0.52 8.2 | 0.56 10.9 | 0.22 11.4
0.20: -0.38 9.9 | -0.34 9.7 | -0.32 10.4 | -0.33 11.3 | -0.27 9.3 | -0.28 9.6 | -0.25 9.0 | -0.28 9.5 | -0.27 8.9 | -0.23 10.2 | -0.28 8.6 | -0.24 10.4 | -0.28 8.8 | -0.25 10.5 | -0.27 8.8
0.00: -0.25 35.8 | -0.24 35.6 | -0.20 34.3 | -0.25 35.9 | -0.24 35.5 | -0.23 34.7 | -0.22 34.7 | -0.30 37.5 | -0.24 35.6 | -0.24 35.7 | -0.21 34.6 | -0.25 35.9 | -0.22 34.6 | -0.34 38.9 | -0.25 35.8
gE (nS)
3.20 3.40 3.60 3.80 4.00 4.20 4.40 4.60 4.80 5.00 5.20 5.40 5.60 5.80 6.00
gI (nS)

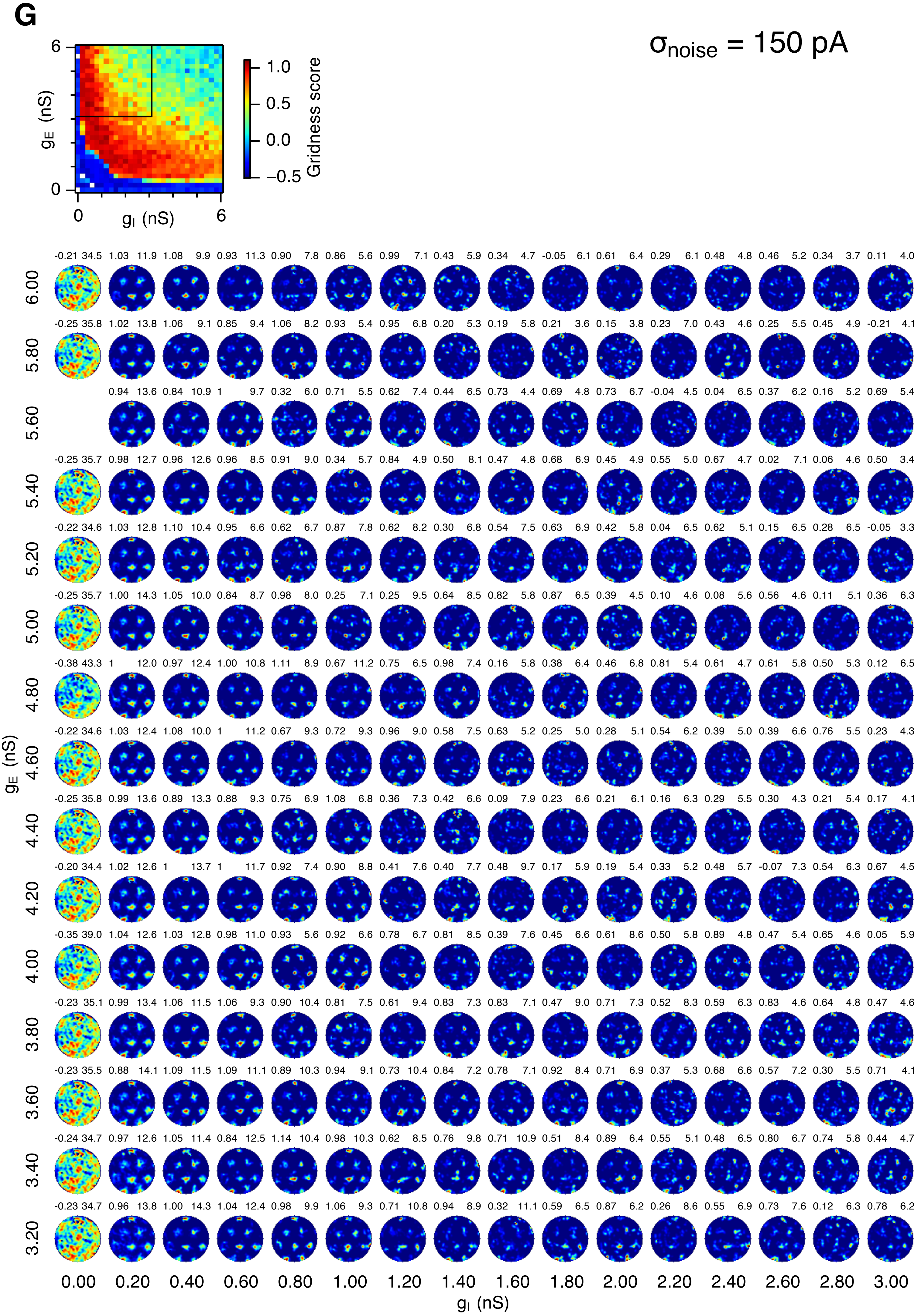
G
σnoise = 150 pA
6
0
gE (nS)
0
gI (nS)
6
1.0
0.5
0.0
−0.5
Gridness score
6.00: -0.21 34.5 1.03 11.9 1.08 9.9 0.93 11.3 0.90 7.8 0.86 5.6 0.99 7.1 0.43 5.9 0.34 4.7 -0.05 6.1 0.61 6.4 0.29 6.1 0.48 4.8 0.46 5.2 0.34 3.7 0.11 4.0
5.80: -0.25 35.8 1.02 13.8 1.06 9.1 0.85 9.4 1.06 8.2 0.93 5.4 0.95 6.8 0.20 5.3 0.19 5.8 0.21 3.6 0.15 3.8 0.23 7.0 0.43 4.6 0.25 5.5 0.45 4.9 -0.21 4.1
5.60: 0.94 13.6 0.84 10.9 1 9.7 0.32 6.0 0.71 5.5 0.62 7.4 0.44 6.5 0.73 4.4 0.69 4.8 0.73 6.7 -0.04 4.5 0.04 6.5 0.37 6.2 0.16 5.2 0.69 5.4
5.40: -0.25 35.7 0.98 12.7 0.96 12.6 0.96 8.5 0.91 9.0 0.34 5.7 0.84 4.9 0.50 8.1 0.47 4.8 0.68 6.9 0.45 4.9 0.55 5.0 0.67 4.7 0.02 7.1 0.06 4.6 0.50 3.4
5.20: -0.22 34.6 1.03 12.8 1.10 10.4 0.95 6.6 0.62 6.7 0.87 7.8 0.62 8.2 0.30 6.8 0.54 7.5 0.63 6.9 0.42 5.8 0.04 6.5 0.62 5.1 0.15 6.5 0.28 6.5 -0.05 3.3
5.00: -0.25 35.7 1.00 14.3 1.05 10.0 0.84 8.7 0.98 8.0 0.25 7.1 0.25 9.5 0.64 8.5 0.82 5.8 0.87 6.5 0.39 4.5 0.10 4.6 0.08 5.6 0.56 4.6 0.11 5.1 0.36 6.3
4.80: -0.38 43.3 1 12.0 0.97 12.4 1.00 10.8 1.11 8.9 0.67 11.2 0.75 6.5 0.98 7.4 0.16 5.8 0.38 6.4 0.46 6.8 0.81 5.4 0.61 4.7 0.61 5.8 0.50 5.3 0.12 6.5
4.60: -0.22 34.6 1.03 12.4 1.08 10.0 1 11.2 0.67 9.3 0.72 9.3 0.96 9.0 0.58 7.5 0.63 5.2 0.25 5.0 0.28 5.1 0.54 6.2 0.39 5.0 0.39 6.6 0.76 5.5 0.23 4.3
4.40: -0.25 35.8 0.99 13.6 0.89 13.3 0.88 9.3 0.75 6.9 1.08 6.8 0.36 7.3 0.42 6.6 0.09 7.9 0.23 6.6 0.21 6.1 0.16 6.3 0.29 5.5 0.30 4.3 0.21 5.4 0.17 4.1
4.20: -0.20 34.4 1.02 12.6 1 13.7 1 11.7 0.92 7.4 0.90 8.8 0.41 7.6 0.40 7.7 0.48 9.7 0.17 5.9 0.19 5.4 0.33 5.2 0.48 5.7 -0.07 7.3 0.54 6.3 0.67 4.5
4.00: -0.35 39.0 1.04 12.6 1.03 12.8 0.98 11.0 0.93 5.6 0.92 6.6 0.78 6.7 0.81 8.5 0.39 7.6 0.45 6.6 0.61 8.6 0.50 5.8 0.89 4.8 0.47 5.4 0.65 4.6 0.05 5.9
3.80: -0.23 35.1 0.99 13.4 1.06 11.5 1.06 9.3 0.90 10.4 0.81 7.5 0.61 9.4 0.83 7.3 0.83 7.1 0.47 9.0 0.71 7.3 0.52 8.3 0.59 6.3 0.83 4.6 0.64 4.8 0.47 4.6
3.60: -0.23 35.5 0.88 14.1 1.09 11.5 1.09 11.1 0.89 10.3 0.94 9.1 0.73 10.4 0.84 7.2 0.78 7.1 0.92 8.4 0.71 6.9 0.37 5.3 0.68 6.6 0.57 7.2 0.30 5.5 0.71 4.1
3.40: -0.24 34.7 0.97 12.6 1.05 11.4 0.84 12.5 1.14 10.4 0.98 10.3 0.62 8.5 0.76 9.8 0.71 10.9 0.51 8.4 0.89 6.4 0.55 5.1 0.48 6.5 0.80 6.7 0.74 5.8 0.44 4.7
3.20: -0.23 34.7 0.96 13.8 1.00 14.3 1.04 12.4 0.98 9.9 1.06 9.3 0.71 10.8 0.94 8.9 0.32 11.1 0.59 6.5 0.87 6.2 0.26 8.6 0.55 6.9 0.73 7.6 0.12 6.3 0.78 6.2
gE (nS)
0.00 0.20 0.40 0.60 0.80 1.00 1.20 1.40 1.60 1.80 2.00 2.20 2.40 2.60 2.80 3.00
gI (nS)

H

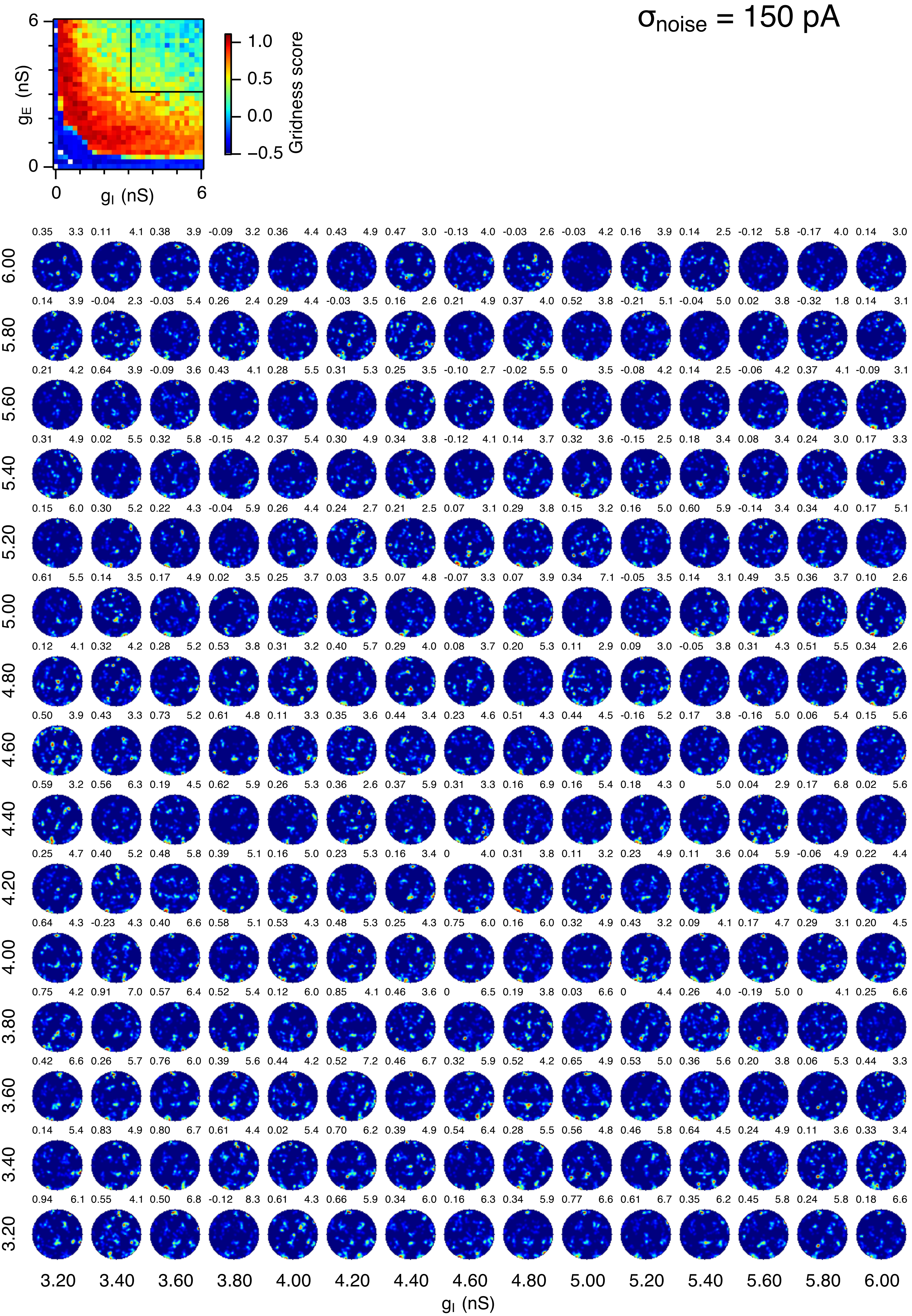

σnoise = 150 pA
6
gE (nS)
0
0
gI (nS)
6
1.0
0.5
0.0
−0.5
Gridness score
0.35 3.3 0.11 4.1 0.38 3.9 -0.09 3.2 0.36 4.4 0.43 4.9 0.47 3.0 -0.13 4.0 -0.03 2.6 -0.03 4.2 0.16 3.9 0.14 2.5 -0.12 5.8 -0.17 4.0 0.14 3.0
6.00
0.14 3.9 -0.04 2.3 -0.03 5.4 0.26 2.4 0.29 4.4 -0.03 3.5 0.16 2.6 0.21 4.9 0.37 4.0 0.52 3.8 -0.21 5.1 -0.04 5.0 0.02 3.8 -0.32 1.8 0.14 3.1
5.80
0.21 4.2 0.64 3.9 -0.09 3.6 0.43 4.1 0.28 5.5 0.31 5.3 0.25 3.5 -0.10 2.7 -0.02 5.5 0 3.5 -0.08 4.2 0.14 2.5 -0.06 4.2 0.37 4.1 -0.09 3.1
5.60
0.31 4.9 0.02 5.5 0.32 5.8 -0.15 4.2 0.37 5.4 0.30 4.9 0.34 3.8 -0.12 4.1 0.14 3.7 0.32 3.6 -0.15 2.5 0.18 3.4 0.08 3.4 0.24 3.0 0.17 3.3
5.40
0.15 6.0 0.30 5.2 0.22 4.3 -0.04 5.9 0.26 4.4 0.24 2.7 0.21 2.5 0.07 3.1 0.29 3.8 0.15 3.2 0.16 5.0 0.60 5.9 -0.14 3.4 0.34 4.0 0.17 5.1
5.20
0.61 5.5 0.14 3.5 0.17 4.9 0.02 3.5 0.25 3.7 0.03 3.5 0.07 4.8 -0.07 3.3 0.07 3.9 0.34 7.1 -0.05 3.5 0.14 3.1 0.49 3.5 0.36 3.7 0.10 2.6
5.00
0.12 4.1 0.32 4.2 0.28 5.2 0.53 3.8 0.31 3.2 0.40 5.7 0.29 4.0 0.08 3.7 0.20 5.3 0.11 2.9 0.09 3.0 -0.05 3.8 0.31 4.3 0.51 5.5 0.34 2.6
4.80
0.50 3.9 0.43 3.3 0.73 5.2 0.61 4.8 0.11 3.3 0.35 3.6 0.44 3.4 0.23 4.6 0.51 4.3 0.44 4.5 -0.16 5.2 0.17 3.8 -0.16 5.0 0.06 5.4 0.15 5.6
4.60
0.59 3.2 0.56 6.3 0.19 4.5 0.62 5.9 0.26 5.3 0.36 2.6 0.37 5.9 0.31 3.3 0.16 6.9 0.16 5.4 0.18 4.3 0 5.0 0.04 2.9 0.17 6.8 0.02 5.6
4.40
0.25 4.7 0.40 5.2 0.48 5.8 0.39 5.1 0.16 5.0 0.23 5.3 0.16 3.4 0 4.0 0.31 3.8 0.11 3.2 0.23 4.9 0.11 3.6 0.04 5.9 -0.06 4.9 0.22 4.4
4.20
0.64 4.3 -0.23 4.3 0.40 6.6 0.58 5.1 0.53 4.3 0.48 5.3 0.25 4.3 0.75 6.0 0.16 6.0 0.32 4.9 0.43 3.2 0.09 4.1 0.17 4.7 0.29 3.1 0.20 4.5
4.00
0.75 4.2 0.91 7.0 0.57 6.4 0.52 5.4 0.12 6.0 0.85 4.1 0.46 3.6 0 6.5 0.19 3.8 0.03 6.6 0 4.4 0.26 4.0 -0.19 5.0 0 4.1 0.25 6.6
3.80
0.42 6.6 0.26 5.7 0.76 6.0 0.39 5.6 0.44 4.2 0.52 7.2 0.46 6.7 0.32 5.9 0.52 4.2 0.65 4.9 0.53 5.0 0.36 5.6 0.20 3.8 0.06 5.3 0.44 3.3
3.60
0.14 5.4 0.83 4.9 0.80 6.7 0.61 4.4 0.02 5.4 0.70 6.2 0.39 4.9 0.54 6.4 0.28 5.5 0.56 4.8 0.46 5.8 0.64 4.5 0.24 4.9 0.11 3.6 0.33 3.4
3.40
0.94 6.1 0.55 4.1 0.50 6.8 -0.12 8.3 0.61 4.3 0.66 5.9 0.34 6.0 0.16 6.3 0.34 5.9 0.77 6.6 0.61 6.7 0.35 6.2 0.45 5.8 0.24 5.8 0.18 6.6
3.20
gE (nS)
3.20 3.40 3.60 3.80 4.00 4.20 4.40 4.60 4.80 5.00 5.20 5.40 5.60 5.80 6.00
gI (nS)

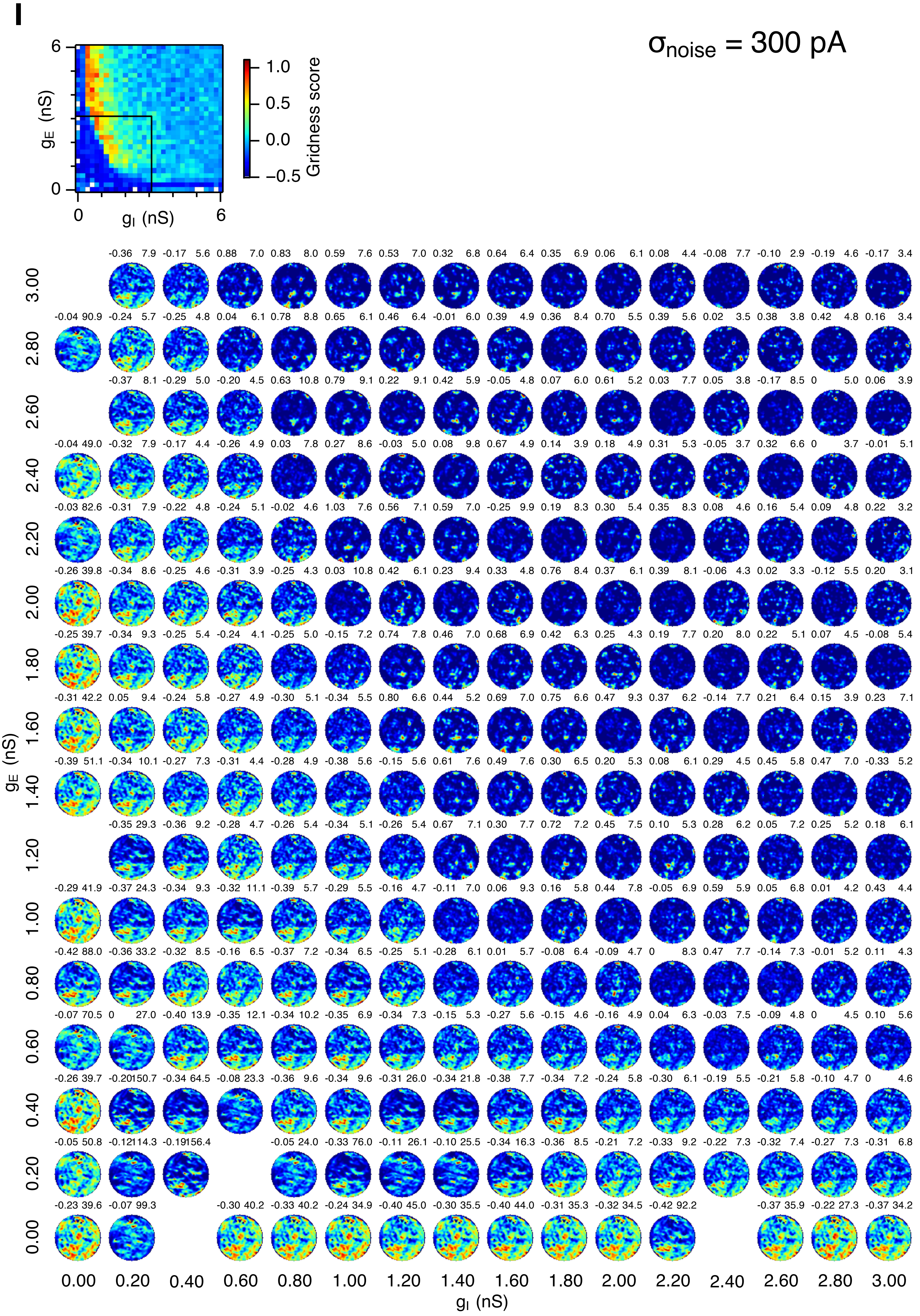
I
σnoise = 300 pA
6
0
gE (nS)
0
gI (nS)
6
1.0
0.5
0.0
−0.5
Gridness score
3.00: -0.36 7.9 -0.17 5.6 0.88 7.0 0.83 8.0 0.59 7.6 0.53 7.0 0.32 6.8 0.64 6.4 0.35 6.9 0.06 6.1 0.08 4.4 -0.08 7.7 -0.10 2.9 -0.19 4.6 -0.17 3.4
2.80: -0.04 90.9 -0.24 5.7 -0.25 4.8 0.04 6.1 0.78 8.8 0.65 6.1 0.46 6.4 -0.01 6.0 0.39 4.9 0.36 8.4 0.70 5.5 0.39 5.6 0.02 3.5 0.38 3.8 0.42 4.8 0.16 3.4
2.60: -0.37 8.1 -0.29 5.0 -0.20 4.5 0.63 10.8 0.79 9.1 0.22 9.1 0.42 5.9 -0.05 4.8 0.07 6.0 0.61 5.2 0.03 7.7 0.05 3.8 -0.17 8.5 0 5.0 0.06 3.9
2.40: -0.04 49.0 -0.32 7.9 -0.17 4.4 -0.26 4.9 0.03 7.8 0.27 8.6 -0.03 5.0 0.08 9.8 0.67 4.9 0.14 3.9 0.18 4.9 0.31 5.3 -0.05 3.7 0.32 6.6 0 3.7 -0.01 5.1
2.20: -0.03 82.6 -0.31 7.9 -0.22 4.8 -0.24 5.1 -0.02 4.6 1.03 7.6 0.56 7.1 0.59 7.0 -0.25 9.9 0.19 8.3 0.30 5.4 0.35 8.3 0.08 4.6 0.16 5.4 0.09 4.8 0.22 3.2
2.00: -0.26 39.8 -0.34 8.6 -0.25 4.6 -0.31 3.9 -0.25 4.3 0.03 10.8 0.42 6.1 0.23 9.4 0.33 4.8 0.76 8.4 0.37 6.1 0.39 8.1 -0.06 4.3 0.02 3.3 -0.12 5.5 0.20 3.1
1.80: -0.25 39.7 -0.34 9.3 -0.25 5.4 -0.24 4.1 -0.25 5.0 -0.15 7.2 0.74 7.8 0.46 7.0 0.68 6.9 0.42 6.3 0.25 4.3 0.19 7.7 0.20 8.0 0.22 5.1 0.07 4.5 -0.08 5.4
1.60: -0.31 42.2 0.05 9.4 -0.24 5.8 -0.27 4.9 -0.30 5.1 -0.34 5.5 0.80 6.6 0.44 5.2 0.69 7.0 0.75 6.6 0.47 9.3 0.37 6.2 -0.14 7.7 0.21 6.4 0.15 3.9 0.23 7.1
1.40: -0.39 51.1 -0.34 10.1 -0.27 7.3 -0.31 4.4 -0.28 4.9 -0.38 5.6 -0.15 5.6 0.61 7.6 0.49 7.6 0.30 6.5 0.20 5.3 0.08 6.1 0.29 4.5 0.45 5.8 0.47 7.0 -0.33 5.2
1.20: -0.35 29.3 -0.36 9.2 -0.28 4.7 -0.26 5.4 -0.34 5.1 -0.26 5.4 0.67 7.1 0.30 7.7 0.72 7.2 0.45 7.5 0.10 5.3 0.28 6.2 0.05 7.2 0.25 5.2 0.18 6.1
1.00: -0.29 41.9 -0.37 24.3 -0.34 9.3 -0.32 11.1 -0.39 5.7 -0.29 5.5 -0.16 4.7 -0.11 7.0 0.06 9.3 0.16 5.8 0.44 7.8 -0.05 6.9 0.59 5.9 0.05 6.8 0.01 4.2 0.43 4.4
0.80: -0.42 88.0 -0.36 33.2 -0.32 8.5 -0.16 6.5 -0.37 7.2 -0.34 6.5 -0.25 5.1 -0.28 6.1 0.01 5.7 -0.08 6.4 -0.09 4.7 0 8.3 0.47 7.7 -0.14 7.3 -0.01 5.2 0.11 4.3
0.60: -0.07 70.5 0 27.0 -0.40 13.9 -0.35 12.1 -0.34 10.2 -0.35 6.9 -0.34 7.3 -0.15 5.3 -0.27 5.6 -0.15 4.6 -0.16 4.9 0.04 6.3 -0.03 7.5 -0.09 4.8 0 4.5 0.10 5.6
0.40: -0.26 39.7 -0.20150.7 -0.34 64.5 -0.08 23.3 -0.36 9.6 -0.34 9.6 -0.31 26.0 -0.34 21.8 -0.38 7.7 -0.34 7.2 -0.24 5.8 -0.30 6.1 -0.19 5.5 -0.21 5.8 -0.10 4.7 0 4.6
0.20: -0.05 50.8 -0.12114.3 -0.19156.4 -0.05 24.0 -0.33 76.0 -0.11 26.1 -0.10 25.5 -0.34 16.3 -0.36 8.5 -0.21 7.2 -0.33 9.2 -0.22 7.3 -0.32 7.4 -0.27 7.3 -0.31 6.8
0.00: -0.23 39.6 -0.07 99.3 -0.30 40.2 -0.33 40.2 -0.24 34.9 -0.40 45.0 -0.30 35.5 -0.40 44.0 -0.31 35.3 -0.32 34.5 -0.42 92.2 -0.37 35.9 -0.22 27.3 -0.37 34.2
gE (nS)
0.00 0.20 0.40 0.60 0.80 1.00 1.20 1.40 1.60 1.80 2.00 2.20 2.40 2.60 2.80 3.00
gI (nS)

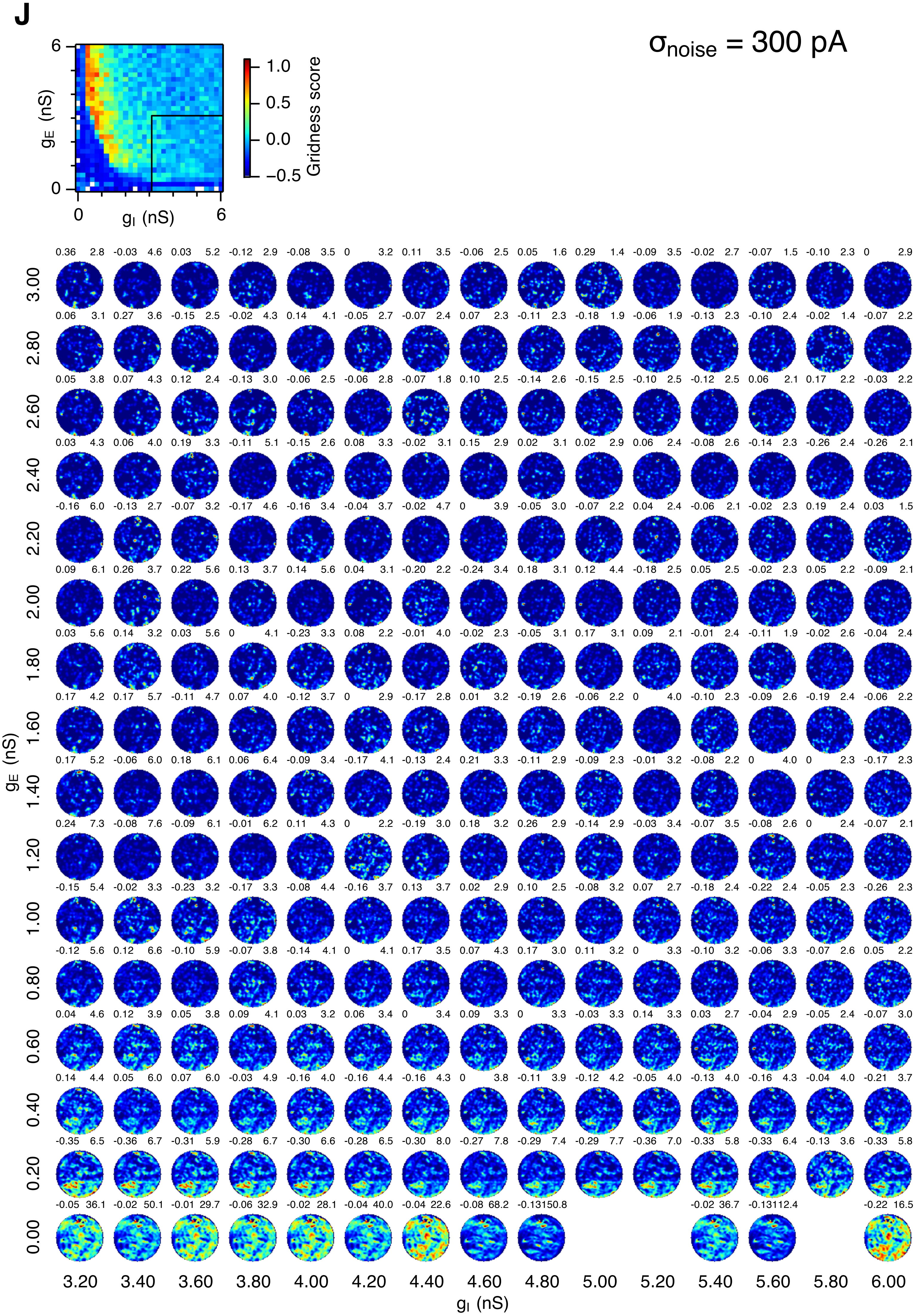

J
σnoise = 300 pA
6
0
gE (nS)
0
gI (nS)
6
1.0
0.5
0.0
−0.5
Gridness score
0.36 2.8 -0.03 4.6 0.03 5.2 -0.12 2.9 -0.08 3.5 0 3.2 0.11 3.5 -0.06 2.5 0.05 1.6 0.29 1.4 -0.09 3.5 -0.02 2.7 -0.07 1.5 -0.10 2.3 0 2.9
3.00
0.06 3.1 0.27 3.6 -0.15 2.5 -0.02 4.3 0.14 4.1 -0.05 2.7 -0.07 2.4 0.07 2.3 -0.11 2.3 -0.18 1.9 -0.06 1.9 -0.13 2.3 -0.10 2.4 -0.02 1.4 -0.07 2.2
2.80
0.05 3.8 0.07 4.3 0.12 2.4 -0.13 3.0 -0.06 2.5 -0.06 2.8 -0.07 1.8 0.10 2.5 -0.14 2.6 -0.15 2.5 -0.10 2.5 -0.12 2.5 0.06 2.1 0.17 2.2 -0.03 2.2
2.60
0.03 4.3 0.06 4.0 0.19 3.3 -0.11 5.1 -0.15 2.6 0.08 3.3 -0.02 3.1 0.15 2.9 0.02 3.1 0.02 2.9 0.06 2.4 -0.08 2.6 -0.14 2.3 -0.26 2.4 -0.26 2.1
2.40
-0.16 6.0 -0.13 2.7 -0.07 3.2 -0.17 4.6 -0.16 3.4 -0.04 3.7 -0.02 4.7 0 3.9 -0.05 3.0 -0.07 2.2 0.04 2.4 -0.06 2.1 -0.02 2.3 0.19 2.4 0.03 1.5
2.20
0.09 6.1 0.26 3.7 0.22 5.6 0.13 3.7 0.14 5.6 0.04 3.1 -0.20 2.2 -0.24 3.4 0.18 3.1 0.12 4.4 -0.18 2.5 0.05 2.5 -0.02 2.3 0.05 2.2 -0.09 2.1
2.00
0.03 5.6 0.14 3.2 0.03 5.6 0 4.1 -0.23 3.3 0.08 2.2 -0.01 4.0 -0.02 2.3 -0.05 3.1 0.17 3.1 0.09 2.1 -0.01 2.4 -0.11 1.9 -0.02 2.6 -0.04 2.4
1.80
0.17 4.2 0.17 5.7 -0.11 4.7 0.07 4.0 -0.12 3.7 0 2.9 -0.17 2.8 0.01 3.2 -0.19 2.6 -0.06 2.2 0 4.0 -0.10 2.3 -0.09 2.6 -0.19 2.4 -0.06 2.2
1.60
0.17 5.2 -0.06 6.0 0.18 6.1 0.06 6.4 -0.09 3.4 -0.17 4.1 -0.13 2.4 0.21 3.3 -0.11 2.9 -0.09 2.3 -0.01 3.2 -0.08 2.2 0 4.0 0 2.3 -0.17 2.3
1.40
0.24 7.3 -0.08 7.6 -0.09 6.1 -0.01 6.2 0.11 4.3 0 2.2 -0.19 3.0 0.18 3.2 0.26 2.9 -0.14 2.9 -0.03 3.4 -0.07 3.5 -0.08 2.6 0 2.4 -0.07 2.1
1.20
-0.15 5.4 -0.02 3.3 -0.23 3.2 -0.17 3.3 -0.08 4.4 -0.16 3.7 0.13 3.7 0.02 2.9 0.10 2.5 -0.08 3.2 0.07 2.7 -0.18 2.4 -0.22 2.4 -0.05 2.3 -0.26 2.3
1.00
-0.12 5.6 0.12 6.6 -0.10 5.9 -0.07 3.8 -0.14 4.1 0 4.1 0.17 3.5 0.07 4.3 0.17 3.0 0.11 3.2 0 3.3 -0.10 3.2 -0.06 3.3 -0.07 2.6 0.05 2.2
0.80
0.04 4.6 0.12 3.9 0.05 3.8 0.09 4.1 0.03 3.2 0.06 3.4 0 3.4 0.09 3.3 0 3.3 -0.03 3.3 0.14 3.3 0.03 2.7 -0.04 2.9 -0.05 2.4 -0.07 3.0
0.60
0.14 4.4 0.05 6.0 0.07 6.0 -0.03 4.9 -0.16 4.0 -0.16 4.4 -0.16 4.3 0 3.8 -0.11 3.9 -0.12 4.2 -0.05 4.0 -0.13 4.0 -0.16 4.3 -0.04 4.0 -0.21 3.7
0.40
-0.35 6.5 -0.36 6.7 -0.31 5.9 -0.28 6.7 -0.30 6.6 -0.28 6.5 -0.30 8.0 -0.27 7.8 -0.29 7.4 -0.29 7.7 -0.36 7.0 -0.33 5.8 -0.33 6.4 -0.13 3.6 -0.33 5.8
0.20
-0.05 36.1 -0.02 50.1 -0.01 29.7 -0.06 32.9 -0.02 28.1 -0.04 40.0 -0.04 22.6 -0.08 68.2 -0.13150.8 -0.02 36.7 -0.13112.4 -0.22 16.5
0.00
gE (nS)
3.20 3.40 3.60 3.80 4.00 4.20 4.40 4.60 4.80 5.00 5.20 5.40 5.60 5.80 6.00
gI (nS)

K

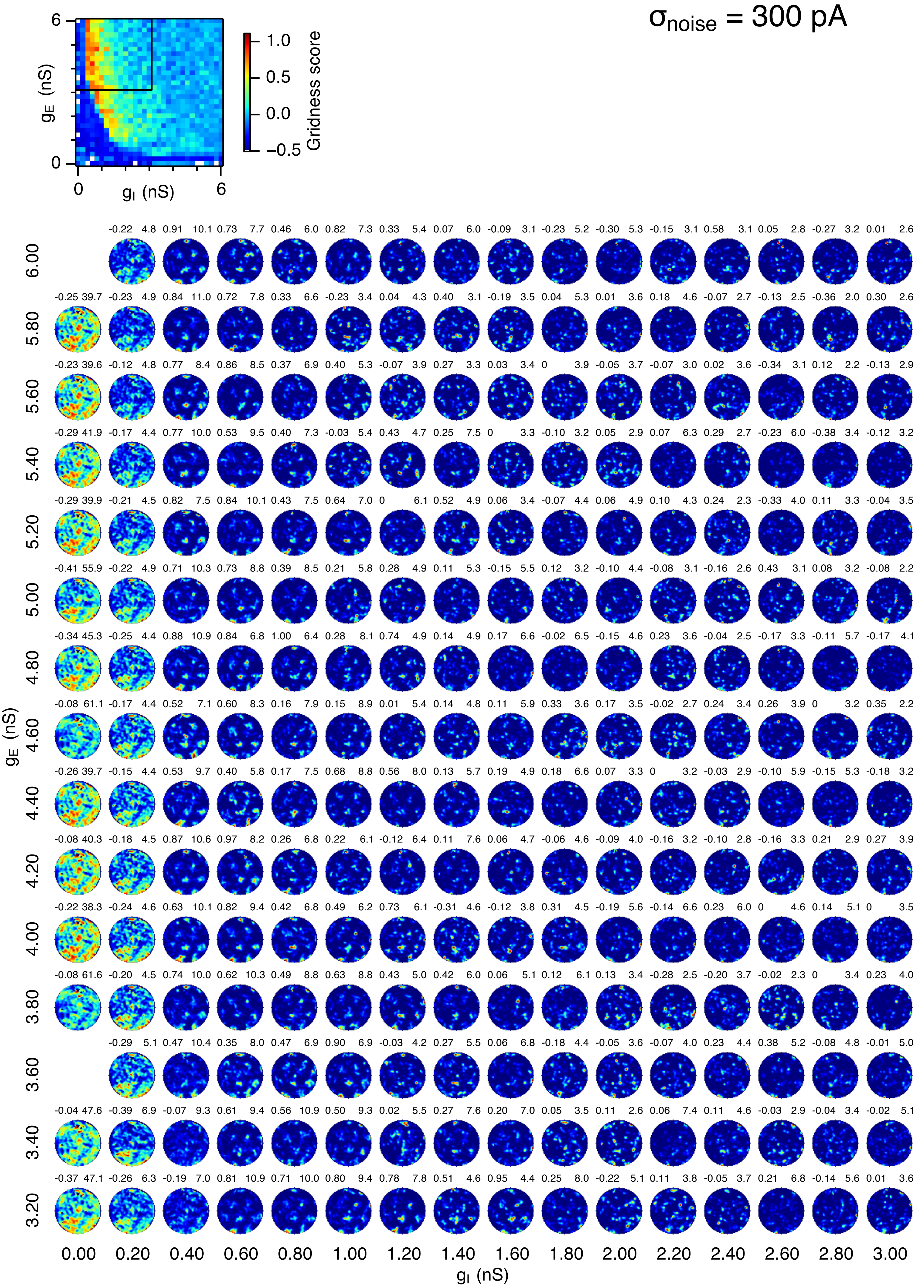

σnoise = 300 pA
6
0
gE (nS)
0
6
gI (nS)
1.0
0.5
0.0
−0.5
Gridness score
-0.22 4.8 0.91 10.1 0.73 7.7 0.46 6.0 0.82 7.3 0.33 5.4 0.07 6.0 -0.09 3.1 -0.23 5.2 -0.30 5.3 -0.15 3.1 0.58 3.1 0.05 2.8 -0.27 3.2 0.01 2.6
-0.25 39.7 -0.23 4.9 0.84 11.0 0.72 7.8 0.33 6.6 -0.23 3.4 0.04 4.3 0.40 3.1 -0.19 3.5 0.04 5.3 0.01 3.6 0.18 4.6 -0.07 2.7 -0.13 2.5 -0.36 2.0 0.30 2.6
-0.23 39.6 -0.12 4.8 0.77 8.4 0.86 8.5 0.37 6.9 0.40 5.3 -0.07 3.9 0.27 3.3 0.03 3.4 0 3.9 -0.05 3.7 -0.07 3.0 0.02 3.6 -0.34 3.1 0.12 2.2 -0.13 2.9
-0.29 41.9 -0.17 4.4 0.77 10.0 0.53 9.5 0.40 7.3 -0.03 5.4 0.43 4.7 0.25 7.5 0 3.3 -0.10 3.2 0.05 2.9 0.07 6.3 0.29 2.7 -0.23 6.0 -0.38 3.4 -0.12 3.2
-0.29 39.9 -0.21 4.5 0.82 7.5 0.84 10.1 0.43 7.5 0.64 7.0 0 6.1 0.52 4.9 0.06 3.4 -0.07 4.4 0.06 4.9 0.10 4.3 0.24 2.3 -0.33 4.0 0.11 3.3 -0.04 3.5
-0.41 55.9 -0.22 4.9 0.71 10.3 0.73 8.8 0.39 8.5 0.21 5.8 0.28 4.9 0.11 5.3 -0.15 5.5 0.12 3.2 -0.10 4.4 -0.08 3.1 -0.16 2.6 0.43 3.1 0.08 3.2 -0.08 2.2
-0.34 45.3 -0.25 4.4 0.88 10.9 0.84 6.8 1.00 6.4 0.28 8.1 0.74 4.9 0.14 4.9 0.17 6.6 -0.02 6.5 -0.15 4.6 0.23 3.6 -0.04 2.5 -0.17 3.3 -0.11 5.7 -0.17 4.1
-0.08 61.1 -0.17 4.4 0.52 7.1 0.60 8.3 0.16 7.9 0.15 8.9 0.01 5.4 0.14 4.8 0.11 5.9 0.33 3.6 0.17 3.5 -0.02 2.7 0.24 3.4 0.26 3.9 0 3.2 0.35 2.2
-0.26 39.7 -0.15 4.4 0.53 9.7 0.40 5.8 0.17 7.5 0.68 8.8 0.56 8.0 0.13 5.7 0.19 4.9 0.18 6.6 0.07 3.3 0 3.2 -0.03 2.9 -0.10 5.9 -0.15 5.3 -0.18 3.2
-0.08 40.3 -0.18 4.5 0.87 10.6 0.97 8.2 0.26 6.8 0.22 6.1 -0.12 6.4 0.11 7.6 0.06 4.7 -0.06 4.6 -0.09 4.0 -0.16 3.2 -0.10 2.8 -0.16 3.3 0.21 2.9 0.27 3.9
-0.22 38.3 -0.24 4.6 0.63 10.1 0.82 9.4 0.42 6.8 0.49 6.2 0.73 6.1 -0.31 4.6 -0.12 3.8 0.31 4.5 -0.19 5.6 -0.14 6.6 0.23 6.0 0 4.6 0.14 5.1 0 3.5
-0.08 61.6 -0.20 4.5 0.74 10.0 0.62 10.3 0.49 8.8 0.63 8.8 0.43 5.0 0.42 6.0 0.06 5.1 0.12 6.1 0.13 3.4 -0.28 2.5 -0.20 3.7 -0.02 2.3 0 3.4 0.23 4.0
-0.29 5.1 0.47 10.4 0.35 8.0 0.47 6.9 0.90 6.9 -0.03 4.2 0.27 5.5 0.06 6.8 -0.18 4.4 -0.05 3.6 -0.07 4.0 0.23 4.4 0.38 5.2 -0.08 4.8 -0.01 5.0
-0.04 47.6 -0.39 6.9 -0.07 9.3 0.61 9.4 0.56 10.9 0.50 9.3 0.02 5.5 0.27 7.6 0.20 7.0 0.05 3.5 0.11 2.6 0.06 7.4 0.11 4.6 -0.03 2.9 -0.04 3.4 -0.02 5.1
-0.37 47.1 -0.26 6.3 -0.19 7.0 0.81 10.9 0.71 10.0 0.80 9.4 0.78 7.8 0.51 4.6 0.95 4.4 0.25 8.0 -0.22 5.1 0.11 3.8 -0.05 3.7 0.21 6.8 -0.14 5.6 0.01 3.6
6.00
5.80
5.60
5.40
5.20
5.00
4.80
4.60
4.40
4.20
4.00
3.80
3.60
3.40
3.20
gE (nS)
0.00 0.20 0.40 0.60 0.80 1.00 1.20 1.40 1.60 1.80 2.00 2.20 2.40 2.60 2.80 3.00
gI (nS)

L

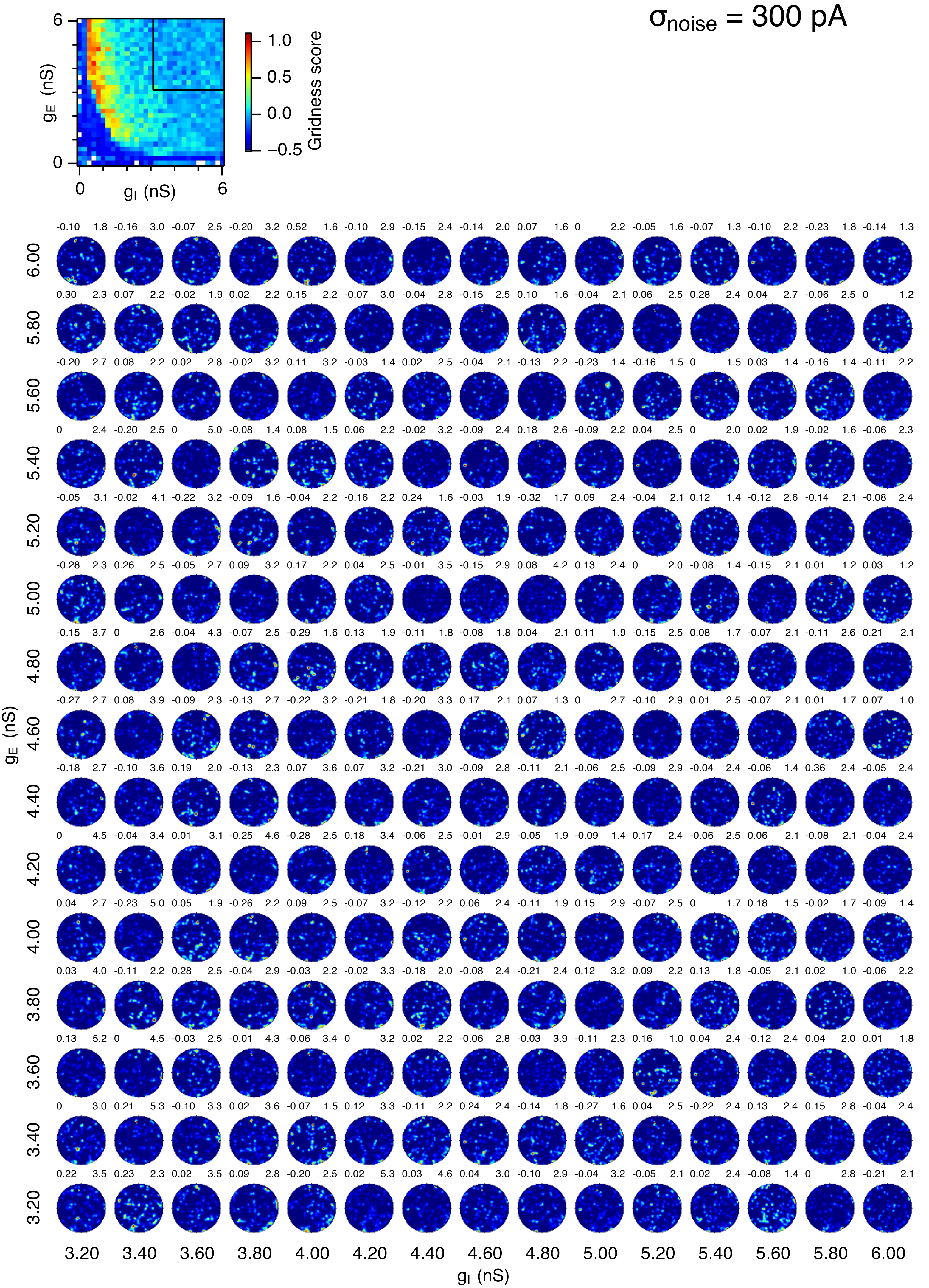

σnoise = 300 pA
6
0
0
6
gE (nS)
gI (nS)
1.0
0.5
0.0
−0.5
Gridness score
gE (nS)
6.00
5.80
5.60
5.40
5.20
5.00
4.80
4.60
4.40
4.20
4.00
3.80
3.60
3.40
3.20
-0.10 1.8 -0.16 3.0 -0.07 2.5 -0.20 3.2 0.52 1.6 -0.10 2.9 -0.15 2.4 -0.14 2.0 0.07 1.6 0 2.2 -0.05 1.6 -0.07 1.3 -0.10 2.2 -0.23 1.8 -0.14 1.3
0.30 2.3 0.07 2.2 -0.02 1.9 0.02 2.2 0.15 2.2 -0.07 3.0 -0.04 2.8 -0.15 2.5 0.10 1.6 -0.04 2.1 0.06 2.5 0.28 2.4 0.04 2.7 -0.06 2.5 0 1.2
-0.20 2.7 0.08 2.2 0.02 2.8 -0.02 3.2 0.11 3.2 -0.03 1.4 0.02 2.5 -0.04 2.1 -0.13 2.2 -0.23 1.4 -0.16 1.5 0 1.5 0.03 1.4 -0.16 1.4 -0.11 2.2
0 2.4 -0.20 2.5 0 5.0 -0.08 1.4 0.08 1.5 0.06 2.2 -0.02 3.2 -0.09 2.4 0.18 2.6 -0.09 2.2 0.04 2.5 0 2.0 0.02 1.9 -0.02 1.6 -0.06 2.3
-0.05 3.1 -0.02 4.1 -0.22 3.2 -0.09 1.6 -0.04 2.2 -0.16 2.2 0.24 1.6 -0.03 1.9 -0.32 1.7 0.09 2.4 -0.04 2.1 0.12 1.4 -0.12 2.6 -0.14 2.1 -0.08 2.4
-0.28 2.3 0.26 2.5 -0.05 2.7 0.09 3.2 0.17 2.2 0.04 2.5 -0.01 3.5 -0.15 2.9 0.08 4.2 0.13 2.4 0 2.0 -0.08 1.4 -0.15 2.1 0.01 1.2 0.03 1.2
-0.15 3.7 0 2.6 -0.04 4.3 -0.07 2.5 -0.29 1.6 0.13 1.9 -0.11 1.8 -0.08 1.8 0.04 2.1 0.11 1.9 -0.15 2.5 0.08 1.7 -0.07 2.1 -0.11 2.6 0.21 2.1
-0.27 2.7 0.08 3.9 -0.09 2.3 -0.13 2.7 -0.22 3.2 -0.21 1.8 -0.20 3.3 0.17 2.1 0.07 1.3 0 2.7 -0.10 2.9 0.01 2.5 -0.07 2.1 0.01 1.7 0.07 1.0
-0.18 2.7 -0.10 3.6 0.19 2.0 -0.13 2.3 0.07 3.6 0.07 3.2 -0.21 3.0 -0.09 2.8 -0.11 2.1 -0.06 2.5 -0.09 2.9 -0.04 2.4 -0.06 1.4 0.36 2.4 -0.05 2.4
0 4.5 -0.04 3.4 0.01 3.1 -0.25 4.6 -0.28 2.5 0.18 3.4 -0.06 2.5 -0.01 2.9 -0.05 1.9 -0.09 1.4 0.17 2.4 -0.06 2.5 0.06 2.1 -0.08 2.1 -0.04 2.4
0.04 2.7 -0.23 5.0 0.05 1.9 -0.26 2.2 0.09 2.5 -0.07 3.2 -0.12 2.2 0.06 2.4 -0.11 1.9 0.15 2.9 -0.07 2.5 0 1.7 0.18 1.5 -0.02 1.7 -0.09 1.4
0.03 4.0 -0.11 2.2 0.28 2.5 -0.04 2.9 -0.03 2.2 -0.02 3.3 -0.18 2.0 -0.08 2.4 -0.21 2.4 0.12 3.2 0.09 2.2 0.13 1.8 -0.05 2.1 0.02 1.0 -0.06 2.2
0.13 5.2 0 4.5 -0.03 2.5 -0.01 4.3 -0.06 3.4 0 3.2 0.02 2.2 -0.06 2.8 -0.03 3.9 -0.11 2.3 0.16 1.0 0.04 2.4 -0.12 2.4 0.04 2.0 0.01 1.8
0 3.0 0.21 5.3 -0.10 3.3 0.02 3.6 -0.07 1.5 0.12 3.3 -0.11 2.2 0.24 2.4 -0.14 1.8 -0.27 1.6 0.04 2.5 -0.22 2.4 0.13 2.4 0.15 2.8 -0.04 2.4
0.22 3.5 0.23 2.3 0.02 3.5 0.09 2.8 -0.20 2.5 0.02 5.3 0.03 4.6 0.04 3.0 -0.10 2.9 -0.04 3.2 -0.05 2.1 0.02 2.4 -0.08 1.4 0 2.8 -0.21 2.1
3.20 3.40 3.60 3.80 4.00 4.20 4.40 4.60 4.80 5.00 5.20 5.40 5.60 5.80 6.00
gI (nS)
